# Gut microbiota associates with frailty in older women

**DOI:** 10.1038/s41467-026-75176-5

**Published:** 2026-07-08

**Authors:** Marina Vilar Geraldi, Chinmay Dwibedi, Raju Jaiswal, Giulia Gregori, Xiaofeng Zhou, Bomin Lv, Yan Zheng, Xiaofeng Wang, Hao Wu, Kristian F. Axelsson, Fredrik Bäckhed, Valentina Tremaroli, Mattias Lorentzon

**Affiliations:** 1 Sahlgrenska Osteoporosis Centre, Department of Internal Medicine and Clinical Nutrition, Institute of Medicine, University of Gothenburg, Gothenburg, Sweden; 2 The Wallenberg Laboratory, Department of Molecular and Clinical Medicine, University of Gothenburg, Gothenburg, Sweden; 3 Department of Clinical Microbiology and Molecular Infection Medicine Sweden (MIMS), Scilifelab, Umeå University, Umeå, Sweden; 4 State Key Laboratory of Genetic and Development of Complex Phenotypes, Human Phenome Institute, and Fudan Microbiome Center, School of Life Sciences, Fudan University, Shanghai, China; 5 National Clinical Research Center for Aging and Medicine, Huashan Hospital, Fudan University, Shanghai, China; 6 Region Västra Götaland, Närhälsan Norrmalm Health Centre, Skövde, Sweden; 7 Region Västra Götaland, Sahlgrenska University Hospital, Department of Clinical Physiology, Gothenburg, Sweden; 8 Novo Nordisk Foundation Microbiome Health Initiative and the National Food Institute, Technical University of Denmark, Kongens Lyngby, Denmark; 9 Department of Internal Medicine, Geriatrics and Emergency Medicine, Sahlgrenska University Hospital, Mölndal, Sweden

**Keywords:** Microbiome, Risk factors

## Abstract

Frailty is a multifactorial geriatric condition linked to increased mortality and adverse health outcomes and is associated with gut microbiome features that differ from those observed in healthy ageing. We analyze gut metagenomic profiles in relation to estimated frailty severity and frailty-related clinical outcomes assessed with an internally developed and validated Frailty Mortality Index (FMI) in the SUPERB cohort, comprising 2,081 Swedish women aged 75–80 years. The FMI is a composite measure that integrates functional, physiological and psychological dimensions associated with frailty and mortality risk, and shows stronger associations with mortality compared to the Charlson Comorbidity Index in the SUPERB cohort. The FMI is inversely associated with microbial diversity, gene richness, and predicted functional capacity, which are linked to physical function, mortality and fall-related injuries. A total of 404 bacterial species are significantly associated with FMI, and most show concordant associations in a Chinese cohort of 1,448 older adults. Here we show microbial signatures linked to frailty and mortality across different continents.

## Abstract (web summary)

In this study the authors identify gut microbiome signatures associated with frailty in older women; these signatures were also linked to physical function, mortality, and fall-related injuries, with many findings replicated in an independent cohort in China.

## Introduction

Frailty is a complex, multifactorial, aging-related syndrome characterized by reduced physiological reserves and increased vulnerability to adverse health outcomes, including falls, hospitalization, disability and mortality^1–5^. Although commonly used tools, such as the Fried Frailty Phenotype^1^, the Rockwood Frailty Index^6^ and the Clinical Frailty Scale, capture specific aspects of frailty^7,8^, existing indices often fail to encompass its full functional, psychological, and physiological dimensions. The Charlson Comorbidity Index (CCI)^9^, while widely adopted for mortality risk stratification, is disease-centric and lacks sensitivity to the broader construct of frailty. To better capture this multidimensional nature, we developed the Frailty Mortality Index (FMI), a composite measure integrating functional and psychosocial aspects in addition to comorbidities. Specifically, the FMI is defined by anthropometrics (age and weight), physical function (walking speed and chair stand), current smoking, mental quality of life (QoL) survey, hospital stay duration, and the CCI.

The gut microbiome is increasingly recognized as a regulator of host physiology and potential contributor to frailty pathophysiology. It influences systemic inflammation, metabolism, musculoskeletal function, and immune and neuroendocrine signaling^10–12^. Aging is accompanied by compositional and functional shifts in the gut microbiome, including reduced diversity, loss of beneficial commensals, and expansion of pro-inflammatory and opportunistic taxa, which have been linked to sarcopenia, cognitive impairment, and multimorbidity^13,14^. While aging alters gut microbiota composition and function, gut microbiome profiles observed in frailty differ from those associated with healthy aging, reflecting not just chronological age but also deterioration of physiological processes^15,16^. Understanding these frailty-associated microbiome features is critical for designing interventions to prevent or delay age-related health deterioration.

In this work, we use metagenomic sequencing to investigate species-level features associated with frailty-related phenotypes captured by the FMI in SUPERB, a large Swedish cohort including 2,081 women aged 75-80 years. We demonstrate that the FMI is more strongly associated with frailty-related clinical outcomes, including injurious falls, hip fractures, and mortality, than the CCI. We further show that higher FMI is associated with reduced microbiota diversity, including lower gene richness and Shannon index. At the species level, FMI is associated with different species in *Enterocloster*, *Clostridium*, *Dysosmobacter* and *Faecalibacterium* in models accounting for the overall decline in microbiome gene richness associated with ageing, thereby distinguishing FMI-associated microbial features from general microbiota decline^17,18^. Several species-level associations identified in SUPERB were replicated in an independent Chinese elderly cohort of 1448 men and women aged 62–96 years, demonstrating concordance across geographically distinct populations.

## Results

### Frailty mortality assessment and study population

**Fig. 1:**
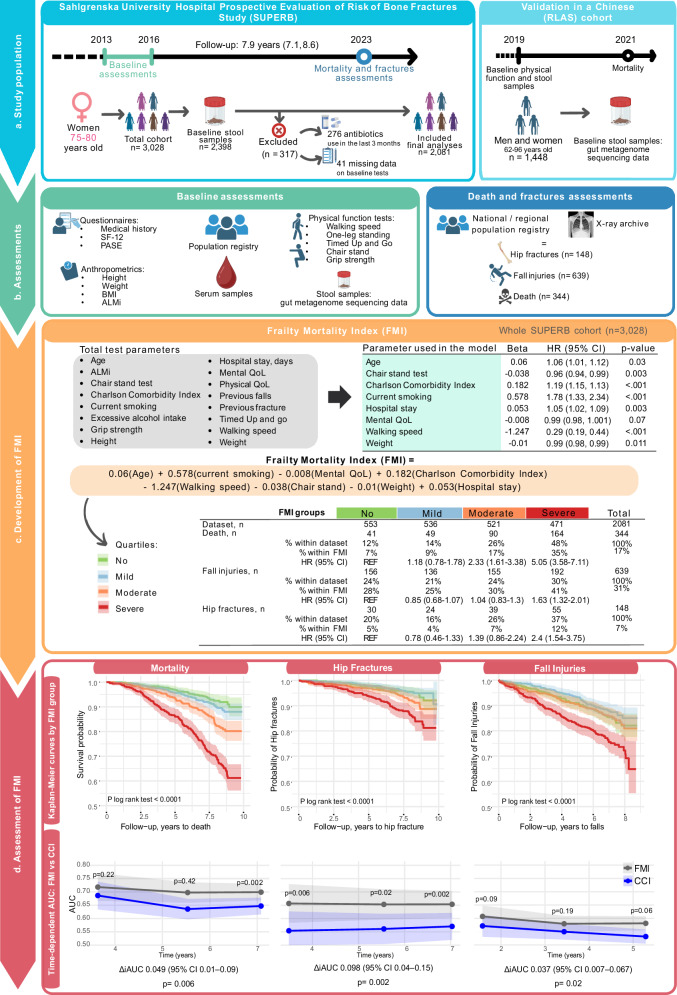
Study population, clinical assessments, and development of the frailty mortality index (FMI). **a** The SUPERB study enrolled 3028 community-dwelling women aged 75–80 years between 2013 and 2016. Baseline assessment included questionnaires, anthropometric, serum sampling, physical function tests, and stool sample collection. Participants were followed for a median of 7.9 years (IQR 7.1–8.6), with mortality, fall injuries, and hip fracture ascertained through 2023. After excluding participants with antibiotic use or missing FMI component data, 2081 participants with available stool metagenomic sequencing data were included. Microbial species associations were evaluated for replication in an independent cohort of older Chinese adults (*n* = 1448; age 62–96 years). **b** Overview of baseline clinical assessments and follow-up outcomes, including death, fall injuries, and hip fractures verified through national and regional registries and hospital X-ray archives. **c**, The FMI was constructed from age, smoking, Charlson Comorbidity Index, hospital stay, mental quality of life, walking speed, chair stand test, and weight. Participants were categorized into four severity groups (no frailty, mild, moderate, and severe frailty). Associations between FMI groups and death, fall injuries, and hip fractures were estimated using Cox proportional hazards models, with no-frailty as the reference. *P*-values were two-tailed and were not adjusted for multiple comparisons. **d** Kaplan–Meier curves stratified by FMI group (top panels) and time-dependent ROC analyses based on Cox proportional hazards models (bottom panels) were used to evaluate associations with mortality, hip fractures, and fall injuries. Solid lines represent estimated survival probabilities or AUC, and shaded bands denote 95% confidence intervals or boostrap confidence intervals. Bootstrap-based comparisons assessed predictive performance of FMI relative to the Charlson Comorbidity Index. ALMi appendicular lean mass index, AUC area under the curve, CI confidence interval, FMI Frailty Mortality Index, HR hazard ratio, iAUC incremental AUC. PASE Physical Activity Scale for the Elderly, QoL quality of life, REF reference, RLAS Rugao Longitudinal Ageing Study, SF-12 12-item Short Form Health Survey. Source data are provided as a Source Data file. Some elements in this figure were created in BioRender. Vilar Geraldi, M. (2026) https://BioRender.com/cre8nv5. The final figure layout was assembled in Affinity Designer 2.

Study participants with complete clinical and metagenomic data (*n* = 2081) were followed for a median (interquartile range) of 7.9 (7.1, 8.6) years (Fig. 1a). We developed the Frailty Mortality Index (FMI), a composite score designed to capture frailty-related mortality risk, in the well-characterized SUPERB cohort (n = 3028) of community-dwelling women aged 75–80 years. Data included assessments of medical history, anthropometrics, physical and mental health, and incidence of injurious falls, hip fractures, and death (Fig. 1b), which are known factors linked to frailty and its outcomes. The FMI was derived from multivariable survival modeling using frailty and mortality associated factors and Cox proportional hazard models with death as the outcome over 7.9-years of median follow-up, as described in methods and in Fig. 1c. The cohort was stratified into quartiles based on FMI scores, with Q1 representing no frailty, Q2 mild frailty, Q3 moderate frailty, and Q4 severe frailty (Fig. 1c). When applied to the analytic subset with complete metagenomic data (*n* = 2081), higher FMI scores were associated with increased risk of mortality, hip fracture, and fall-related injuries (Fig. 1c). Compared to the non-frail group, individuals in the severe FMI group had 5 times higher risk of death (hazard ratio [HR] 5.05, 95% CI 3.58–7.11, *P* < 0.001), a higher risk of injurious falls (HR 1.63, 95% CI 1.32–2.01, *P* < 0.001), and more than twofold higher risk of hip fracture (HR 2.4, 95% CI 1.54–3.75, *P* < 0.001) (Fig. 1c). Progressively higher risks of mortality, hip fractures, and fall injuries were observed across increasing FMI groups. Bootstrap-based comparisons of time-dependent AUCs demonstrated improved predictive performance of the FMI compared with the CCI (Fig. 1d).

Clinical and functional characteristics across FMI groups are presented in Table 1, and their distribution by FMI is shown in Supplementary Fig. 1. The severe FMI group was associated with older age, a higher prevalence of current smoking, elevated CCI, and worse physical function, as reflected by reduced grip strength, slower walking speed, and fewer chair stands, alongside lower physical and mental quality of life scores, defined by the Short Form survey-12 (SF-12) questionnaire (Table 1). Furthermore, increasing FMI severity was significantly associated with systemic inflammation, measured by the Systemic Inflammation Response Index (SIRI), as well as with more prevalent medication use, including statins, proton pump inhibitors and metformin (Table 1).

**Table 1:** Baseline characteristics according to groups of Frailty Mortality Index

Groups of Frailty Mortality Index	No	Mild	Moderate	Severe	P
Number of subjects	553	536	521	471	
Frailty Mortality Index	−1.0 [-1.28;−0.83]^A^	−0.44 [−0.57;−0.30]^B^	0.14 [−0.02;0.29]^C^	1.14 [0.75;1.78]^D^	<0.001
Age, years	76.7 [75.72;77.82]^A^	77.5 [76.45;79.00] ^B^	78.1 [76.91;79.52] ^C^	78.5 [77.10;79.75] ^C^	<0.001
Body mass index, kg/m^2^	25.5 [22.99;28.23] ^A^	25.6 [23.16;28.46] ^AB^	26.0 [23.49;28.78] ^AB^	26.3 [23.11;30.25] ^B^	0.02
Current smoking, n (%)	1 (0.18)	6 (1.12)	24 (4.61)	68 (14.41)	<0.001
High alcohol intake, n (%)	3 (0.54)	0 (0.00)	2 (0.38)	4 (0.85)	0.18
Physical Activity Scale for Elderly *	114.3 [82.46;153.95] ^A^	106.5 [75.71;141.00] ^B^	91.4 [65.00;126.25] ^C^	75.4 [52.14;107.23] ^D^	<0.001
Physical component score SF-12	54.8 [47.39;56.58] ^A^	51.0 [43.10;55.50] ^B^	45.6 [35.26;54.11] ^C^	36.3 [29.47;46.85] ^D^	<0.001
Mental component score SF-12	57.9 [53.86;60.74] ^A^	57.5 [52.47;60.61] ^A^	55.9 [48.28;59.72] ^B^	50.8 [41.02;58.26] ^C^	<0.001
Education level	4.0 [3.0;6.0] ^A^	3.0 [2.0;5.0] ^B^	3.0 [1.0;5.0] ^B^	3.0 [1.0;5.0] ^B^	<0.001
Appendicular Lean Mass index, kg/m^2^ *	6.4 [5.85;6.85] ^A^	6.2 [5.72;6.78] ^AB^	6.1 [5.71;6.77] ^B^	6.2 [5.71;6.87] ^AB^	0.004
Glycated hemoglobin (HbA1c), % ‡	36.0 [35.0;38.0] ^A^	37.0 [35.0;40.0] ^AB^	37.0 [35.0;41.0] ^B^	38.0 [36.0;44.0] ^B^	<0.001
Vitamin D (nmol/L) *	61.1 [49.20;74.60] ^A^	61.7 [48.00;75.48] ^A^	59.7 [48.05;74.10] ^A^	59.7 [46.88;76.75] ^A^	0.29
Albumin (g/dL) *	4.33 [4.17;4.49] ^A^	4.30 [4.11;4.49] ^AB^	4.27 [4.07;4.47] ^B^	4.27 [4.08;4.47] ^B^	<0.001
Estimated Glomerular Filtration Rate (mL/min) *	60.0 [51.82;68.31] ^A^	58.3 [50.41;67.81] ^AB^	57.4 [48.92;67.10] ^B^	56.5 [47.30;68.00] ^B^	0.002
Hemoglobin (g/dL) †	13.7 [13.1;14.3] ^A^	13.6 [13.0;14.2] ^A^	13.6 [12.8;14.3] ^AB^	13.4 [12.6;14.2] ^B^	0.001
Charlson Comorbidity Index	0.0 [0.0;0.0]	0.0 [0.0;1.0]	1.0 [0.0;2.0]	2.0 [1.0;3.0]	<0.001
0	462 (83.5)	344 (64.2)	213 (40.9)	78 (16.5)	<0.001
1	64 (11.6)	116 (21.6)	149 (28.6)	107 (22.7)	<0.001
2	25 (4.5)	70 (13.1)	123 (23.6)	132 (28.0)	<0.001
3	2 (0.4)	6 (1.1)	23 (4.4)	80 (17.0)	<0.001
≥4	0 (0.0)	0 (0.0)	13 (2.5)	75 (15.9)	<0.001
Systemic Inflammation Response Index (SIRI) †	0.63 [0.44;0.88] ^A^	0.69 [0.46;0.97] ^A^	0.77 [0.54;1.09] ^B^	0.87 [0.56;1.26] ^C^	<0.001
Gene Richness (x10^6^)	1.40 (1.20, 1.57) ^A^	1.34 (1.16, 1.52) ^A^	1.29 (1.07, 1.47) ^B^	1.21 (1.01, 1.38) ^C^	<0.001
*Medications*					
Statin, n (%)	98 (17.7)	135 (25.2)	128 (24.6)	158 (33.5)	<0.001
Metformin, n (%)	9 (1.6)	21 (3.9)	33 (6.3)	38 (8.1)	<0.001
Proton pump inhibitors, n (%)	42 (7.6)	62 (11.6)	72 (13.8)	109 (23.1)	<0.001
Fall no fracture, n (%)	74 (13.4)	67 (12.5)	75 (14.4)	105 (22.3)	<0.001
Fall the past year, n (%)	118 (21.3)	126 (23.5)	147 (28.2)	186 (39.4)	<0.001
*Physical function tests*					
Grip strength, kg †	17.8 [14.5;21.0] ^A^	16.0 [12.0;19.0] ^B^	14.0 [11.0;17.5] ^C^	12.0 [8.0;16.0] ^D^	<0.001
Timed Up and Go, s *	6.8 [6.1;7.5] ^A^	7.5 [6.7;8.4] ^B^	8.4 [7.4;9.4] ^C^	10.1 [8.5;13.3] ^D^	<0.001
Walking speed, m/s	1.49 [1.40;1.59] ^A^	1.34 [1.24;1.42] ^B^	1.21 [1.11;1.32] ^C^	1.02 [0.86;1.15] ^D^	<0.001
Chair stand, n/30 s	13.0 [12.0;15.0] ^A^	12.0 [10.0;13.0] ^B^	10.0 [9.0;12.0] ^C^	8.0 [3.8;10.0] ^D^	<0.001
One leg standing, s §	17.3 [8.0;27.9] ^A^	12.4 [6.2;22.6] ^B^	10.1 [5.3;19.4] ^C^	6.9 [3.9;15.3] ^D^	<0.001
*Butyrate pathways*					
*but*	3346.0 [2746.0;3894.0] ^A^	3322.0 [2793.8;3935.8] ^A^	3273.0 [2713.0;3826.0] ^AB^	3152.5 [2590.5;3790.5] ^B^	0.003
*buk*	3825.0 [3276.0;4496.0]	3869.5 [3233.5;4571.3]	3898.0 [3223.0;4581.0]	3938.0 [3388.5;4611.8]	0.28
*atoA/D*	1035.0 [756.0;1396.0] ^A^	1063.0 [777.5;1451.3] ^AB^	1042.0 [745.0;1420.0] ^AB^	1121.5 [777.0;1671.0] ^B^	0.02
*4hbt*	1938.0 [1356.0;2654.0]	1928.0 [1375.0;2598.3]	1807.0 [1352.0;2451.0]	1924.5 [1390.0;2556.0]	0.24
Total butyrate production potential	10281.0 [9241.0;11807.0]	10441.5 [9161.0;11761.3]	10095.0 [9056.0;11649.0]	10347.0 [9152.5;11968.5]	0.22

Data are shown as number (%) for dichotomous variables and as median [interquartile range] for continuous, non-normally distributed variables. Overall differences among the four frailty groups were tested with the Kruskal–Wallis test (continuous variables) or the χ^2^ (dichotomous variables). When the Kruskal–Wallis test was significant, Dunn’s post-hoc test with Bonferroni correction was applied. Frailty groups that share the same letter are not significantly different (*P* ≥ 0.05); groups with different letters are significantly different (*P* < 0.05). Total *N* = 2081. All p-values were two-sided.

Missing individuals:

*Less than 10,

†36–80,

§364,

‡802

### Associations between FMI and microbiome diversity

**Fig. 2:**
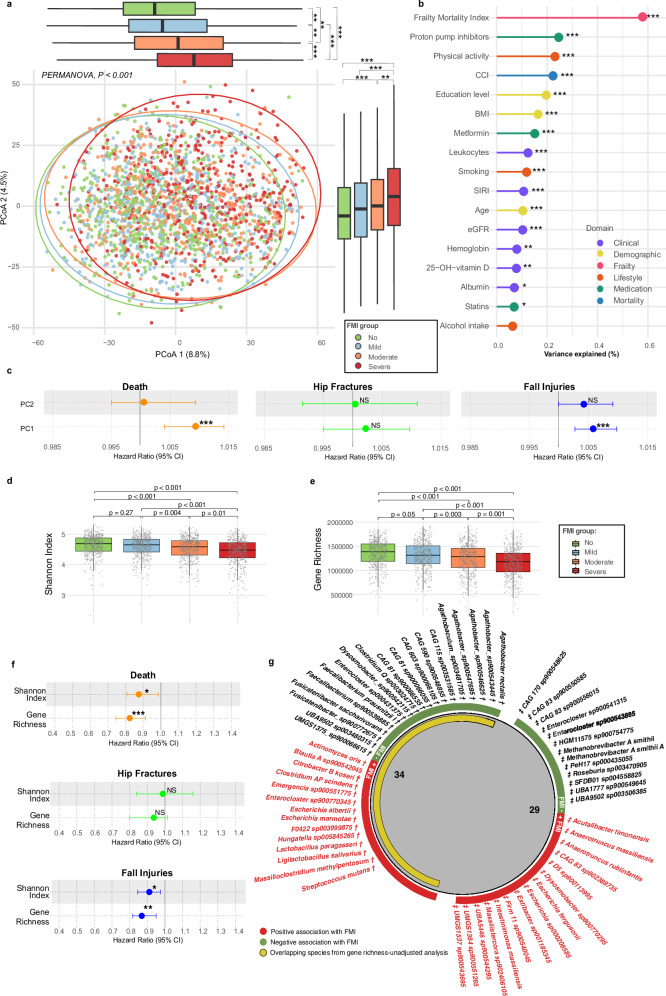
Associations between frailty mortality index (FMI) and gut microbiome diversity. **a** Principal coordinate analysis (PCoA) of species-level Aitchison distances showing gut microbial composition by FMI severity group. Box plots along PCoA1 and PCoA2 show the distribution of individual scores within each group. **b** Proportion of compositional variance explained by FMI and other domains, including demographics, clinical, medication, mortality, and lifestyle variables. **c** Cox models showing associations of PCoA1 and PCoA2 scores with mortality, hip fractures, and fall injuries. Points represent hazard ratios and error bars denote 95% confidence intervals. Box plot showing the distribution of the Shannon index (**d**) and gene richness (**e**) across FMI groups. **f** Cox models showing associations of Shannon diversity index and gene richness with mortality, hip fracture, and fall injuries. Points represent hazard ratios and error bars denote 95% confidence intervals. **g** Venn diagram of species significantly associated with FMI after Bonferroni correction, before and after adjustment for gene richness. Species uniquely identified after gene richness adjustment are shown with the direction of association. All regression and Cox models were adjusted for age, BMI, education, smoking status, alcohol intake, statin use, proton pump inhibitor use, and metformin use. *P* values were calculated using the Kruskal-Wallis test and corrected for multiple comparisons using Bonferroni. Box plots show medians, interquartile ranges, and whiskers extending to 1.5 × the interquartile range. *N* = 2081. ***P ≤ 0.001; ** 0.001 < *P* ≤ 0.01; * *P* < 0.05; NS, not significant. *CCI* Charlson Comorbidity Index, *CI* confidence interval, *eGFR* estimated glomerular filtration rate, *FMI* Frailty Mortality Index, *PCoA* principal coordinate, *SIRI* systemic inflammation response index; † species that remained statistically significant after adjustment for gene richness; ‡ unique species identified after gene richness adjustment. Source data are provided as a Source Data file.

The gut microbiota characteristics of the study population revealed significant differences in microbial composition, diversity, and gene richness across varying levels of FMI (Fig. 2a–g). Distinct clustering of gut microbial compositions among the four FMI groups (no, mild, moderate, severe) was observed in the principal coordinate analysis (PCoA) based on species-level Aitchison dissimilarity (Fig. 2a). The first principal coordinate explained 8.8% of the total compositional variance, significantly separating all FMI groups, whilst the second principal coordinate accounted for 4.5% of the variance, further differentiating the moderate and severe groups (*P* < 0.001) from the no FMI group, highlighting the progressive shift in microbial profiles with increasing FMI (Fig. 2a). In multivariable analysis, FMI explained 0.6% of the total gut microbiota compositional variation (R² = 0.006, *P* = 0.001, PERMANOVA, 999 permutations). Sensitivity analysis using Bray-Curtis dissimilarity (Supplementary Fig. 2) confirmed significant compositional differences across FMI groups (R² = 0.007, *P* = 0.001). In comparison, all other tested factors, such as body mass index (BMI), medication use, physical activity, smoking, education level, and the CCI, each explained considerably smaller proportions, with the CCI accounting for only 0.2% of the variation (Fig. 2b; Supplementary Figs. 2 and 3). The gut microbiota-derived first principal coordinate was associated with mortality risk and fall injuries (Fig. 2c).

Alpha diversity, as determined by the Shannon index, and gene richness, reflecting the total number of unique predicted genes, were significantly associated with FMI groups and decreased with increasing frailty in the FMI groups (Fig. 2d, e). We examined whether overall microbiome diversity and functional richness were associated with frailty-related clinical outcomes (Fig. 2f). Higher Shannon diversity and gene richness were significantly associated with a lower risk of both fall injury and death, whereas no significant association was observed for hip fracture (Fig. 2f). These findings indicate that reductions in gut microbial diversity and functional gene content may reflect impaired ecological resilience of the microbiome, and are associated with vulnerability to frailty-related adverse events.

### Microbial taxonomic shifts associated with FMI, comorbidities and medication use

To identify bacterial species significantly associated with FMI severity, we performed linear regression analyses using the main model, adjusting for age, BMI, education level, smoking status, alcohol intake, and use of statins, proton pump inhibitors (PPI), and metformin. A gene richness adjusted model included additional adjustment for gene richness to reveal associations specific to FMI and not secondary to the decreasing diversity observed in the FMI groups (Fig. 2e). In the main model, without adjusting for gene richness, 404 species were significantly associated with FMI after correcting for multiple testing using a Bonferroni-adjusted threshold (α = 0.05/2057; *P* < 2.43 × 10⁻⁵), whereas 63 species were significant when additionally adjusting for gene richness (Fig. 2g; Supplementary Data 1). 34 species were significantly associated with FMI both before and after adjustment for gene richness, including several species in the genus *Enterocloster* and oral bacteria and pathogens (e.g., *Streptococcus mutans* and *Actinomyces oris*; Fig. 2g). 29 species (e.g., *Massilistercora* sp902406105, *Dysosmobacter* sp900770295, *Acutalibacter timonensis*, *Anaerotruncus rubiinfantis*, and *Roseburia* sp003470905) emerged as significant only after adjusting for gene richness (Fig. 2g), representing additional intrinsic associations with FMI.

To distinguish microbiome features associated with frailty from those related to comorbid disease burden and baseline health status, we compared microbial associations with the FMI and the CCI and performed additional sensitivity analyses. In linear regression models adjusted for age, BMI, education, smoking, alcohol intake, and use of statins, proton pump inhibitors, and metformin, 82 species were significantly associated with the CCI after Bonferroni correction (*P* < 2.43 × 10⁻⁵). 80 of these species overlapped with those associated with the FMI (Supplementary Data 2).

To further address potential confounding by comorbidities, we conducted a complementary sensitivity analysis (Supplementary Data 3). In this comorbidity-adjusted model, we additionally controlled for baseline diseases, including rheumatoid arthritis, dementia, ischemic heart disease, heart failure, cerebrovascular disease, chronic pulmonary disease, liver disease, diabetes mellitus, renal failure, hemiplegia or paraplegia, peptic ulcer disease, solid non-metastatic tumor, lymphoma or leukemia, hyperparathyroidism, hyperthyroidism, and malnutrition. After adjustment, all FMI-associated species remained significant.

In addition to including medication use as covariates in all models, we also performed stratified analyses by PPI, statin, and metformin use (users vs. non-users). The FMI–microbiome associations showed near-complete directional consistency (>98.5%) across medication strata, with 77%, 59.4%, and 18.6% of associations remaining significant in statins, PPI, and metformin users, respectively (Supplementary Data 4). The number of significant associations exceeded that expected by chance under permutation analyses for PPI and statins, whereas fewer were observed among metformin users, which had the smallest sample size (*n* = 101). This likely reflects reduced statistical power in smaller subgroups, particularly among metformin users (*n* = 101; PPI users *n* = 285; and statins users *n* = 519), as well as higher FMI levels in medication groups, indicating greater frailty severity.

### FMI-associated microbiome and clinical outcomes

**Fig. 3:**
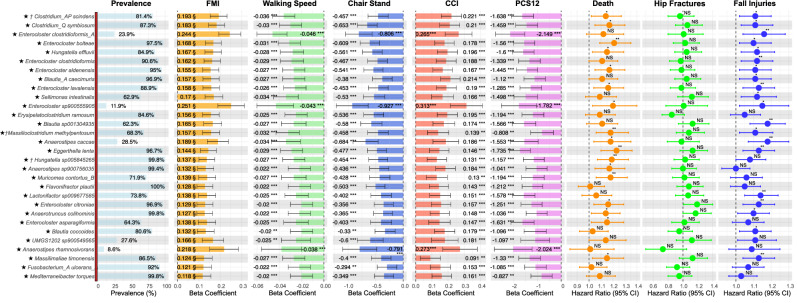
Microbial species positively associated with Frailty Mortality Index and clinical outcomes. Association of bacterial species with FMI, physical function measures (chair stand, and walking speed), Charlson Comorbidity Index, physical quality-of-life scores (PCS-12), and risks of mortality, hip fractures, and fall injuries. Beta coefficients (95% CI) from linear regression models are unstandardized, and species prevalence is indicated as the percentage of participants carrying each taxon. Cox proportional hazards models were used to estimate hazard ratios (95% CI) for clinical outcomes. Colored bars indicate adjusted beta coefficients, points represent hazard ratios, and error bars denote 95% confidence intervals. *N* = 2081. Analyses were adjusted for age, *BMI*, education level, smoking status, alcohol intake, statin use, proton pump inhibitor use, and metformin use. *CCI* Charlson Comorbidity Index, *CI* confidence interval, *FMI* Frailty Mortality Index, *PCS12* Physical Component Score; §Significant FMI species at Bonferroni-corrected threshold (α = 0.05/2057; *P* < 2.43 × 10⁻⁵); ★ Species that remained significantly associated with FMI when adjusting for comorbidities; ***P < 0.00012 (Bonferroni-adjusted for 404 tests); **FDR < 0.05; *FDR < 0.1; NS, not significant. Source data are provided as a Source Data file.

Species with a prevalence ≥15%, that were most significantly and positively associated with FMI included *Enterocloster* spp., *Clostridium Q symbiosum*, *Clostridium A scindens, Erysipelatoclostridium ramosum* and *Eggerthella lenta*. Notably, several of these species demonstrated strong positive associations with FMI and frailty-related parameters (Fig. 3 and Supplementary Data 2) and were associated with reduced physical and mental function and increased risk of injurious falls and death (Fig. 3; Supplementary Data 5 and 6).

**Fig. 4:**
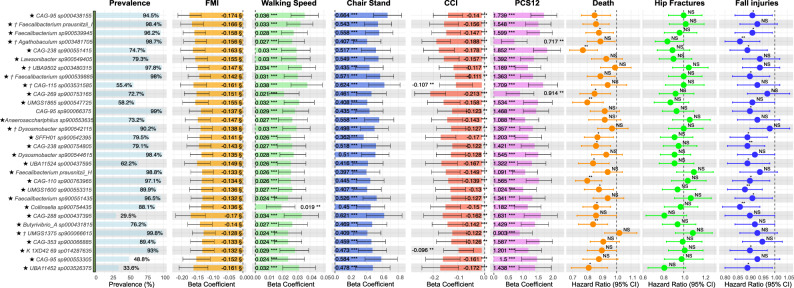
Microbial species negatively associated with Frailty Mortality Index and clinical outcomes. Association of bacterial species with FMI, physical function measures (chair stand and walking speed), Charlson Comorbidity Index, physical quality-of-life scores (PCS-12), and risks of mortality, hip fractures, and fall injuries. Beta coefficients (95% CI) from linear regression models are unstandardized, and species prevalence is indicated as the percentage of participants carrying each taxon. Cox proportional hazards models were used to estimate hazard ratios (95% CI) for clinical outcomes. Colored bars indicate adjusted beta coefficients, points represent hazard ratios, and error bars denote 95% confidence intervals. *N* = 2081. Analyses were adjusted for age, BMI, education level, smoking status, alcohol intake, statin use, proton pump inhibitor use, and metformin use. CCI Charlson Comorbidity Index, CI confidence interval, FMI Frailty Mortality Index, PCS12 Physical Component Score; §Significant FMI species at Bonferroni-corrected threshold (α = 0.05/2057; *P* < 2.43 × 10⁻⁵); ★ Species that remained significantly associated with FMI when adjusting for comorbidities; ***P < 0.00012 (Bonferroni-adjusted for 404 tests); ** FDR < 0.05; * FDR < 0.1; NS not significant. Source data are provided as a Source Data file.

High-prevalence (≥15%) species that were most significantly negatively associated with FMI included many uncharacterized metagenome-assembled genomes (MAGs) and potential butyrate producers, such as *Faecalibacterium prausnitzii* clades I and H and *Faecalibacterium* sp900539945, as well as *Dysosmobacter* sp900544615 and *Agathobaculum* sp003481705 (Fig. 4 and Supplementary Data 7). These species were associated with better physical and mental function and increased survival (Fig. 4, Supplementary Data 7 and 8).

Associations between microbial species and clinical or biochemical parameters are summarized in Supplementary Fig. 4, highlighting consistent patterns between taxa linked to frailty and host phenotypes. Species positively associated with FMI were generally negatively associated with physical activity and positively associated with SIRI, age, and medication use. In contrast, species negatively associated with FMI tended to correlate positively with physical activity and negatively with SIRI, age, and medication use (Supplementary Fig. 4).

### Gut microbiota metabolic potential, FMI and clinical outcomes

To investigate the metabolic potential of the gut microbiota associated with FMI and further characterize the species-level associations, we quantified gut metabolic modules (GMMs), representing manually curated metabolic pathways in the human gut microbiota^19^, and assessed their associations with FMI (Supplementary Data 9).

**Fig. 5:**
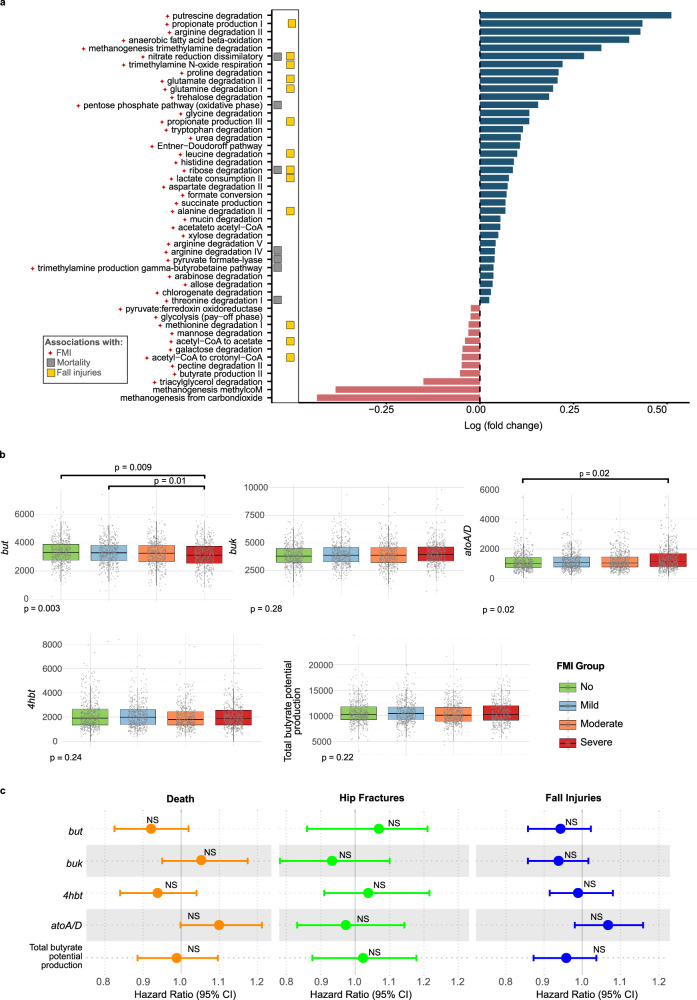
Functional and butyrate production potential linked to the Frailty Mortality Index (FMI) and clinical outcomes. **a**, Differential abundance analysis of gut metabolic modules (GMMs) comparing participants with no frailty versus severe FMI. Modules shown are those differentially abundant (*q* < 0.05), expressed as natural log-transformed fold changes. Positive log-fold changes indicate higher abundance in severe FMI. Red ✦ highlight GMM associated with FMI in Spearman correlation analysis. Grey labels represent modules associated with mortality, while yellow labels indicate modules associated with injurious falls in Cox proportional hazard models (*q* < 0.05). **b** Box plots displaying the distribution (median, interquartile range, and whiskers extending 1.5 × interquartile range) of butyrate production potential across FMI severity groups. *P* values were calculated using the Kruskal-Wallis test and corrected for multiple comparisons using Bonferroni. **c** Forest plot showing associations between butyrate production potential and risk of mortality, hip fractures, and fall injuries from Cox proportional hazards models. Models were adjusted for age, BMI, education level, smoking status, alcohol intake, statin use, proton pump inhibitor use, and metformin use. Points represent hazard ratios and error bars denote 95% confidence intervals. The *P* values were two-tailed; no adjustments were made for multiple comparisons. *N* = 2081. CI confidence interval, FMI Frailty Mortality Index; *P < 0.05; NS not significant. Source data are provided as a Source Data file.

GMMs most strongly and positively associated with FMI (no vs. severe) included functions involved in amino acid degradation, particularly glutamine, glutamate and arginine, as well as ribose catabolism, lactate metabolism and propionate production, trimethylamine/trimethylamine-N-oxide metabolism, and anaerobic respiration including nitrate reduction (Fig. 5a and Supplementary Data 9). Conversely, GMMs for methanogenesis from carbon dioxide, triacylglycerol degradation, cysteine biosynthesis/homocysteine degradation, pectin degradation, acetate production and butyrate production II, corresponding to the butyryl-CoA CoA-transferase pathway and the *but* terminal gene, were among the most strongly and inversely associated with FMI (Fig. 5a). Several of these modules were also associated with mortality and injurious falls (Fig. 5a and Supplementary Data 9). All GMMs significantly associated with FMI (no vs. severe) remained significant after adjusting for gene richness.

To follow up on the associations of species profiles and GMMs with FMI, we next quantified the relative abundance of terminal genes involved in butyrate production from carbohydrates (*but* and *buk*) and proteins (*atoA/D* and *4hbt*). The analyses showed a significant decrease in *but* abundance across the FMI severity groups and an increase in *atoA/D* in the severe group compared with no frailty (Fig. 5b). Several species positively associated with FMI, such as *Enterocloster* spp.*, Blautia* spp.*, Sellimonas intestinalis, Hungatella effluvii*, and *Eggerthella lenta*, were negatively associated with predicted total butyrate production capacity and/or *but* abundance (Supplementary Fig. 5a and Supplementary Data 10), while species negatively associated with FMI, such as *F. prausnitzii*, *Anaerosacchariphilus* sp900553635, *Dysosmobacter* sp900544615 and several MAGs, were positively associated with the *but* pathway or predicted total butyrate production capacity (Supplementary Fig. 5b and Supplementary Data 11).

However, Cox proportional hazards revealed no significant associations between butyrate-production pathways per se and the risk of mortality, hip fracture, or fall injuries (Fig. 5c and Supplementary Data 12). These results suggest that features beyond butyrate-production capacity may contribute to the observed associations with low frailty, potentially including other functional properties, such as anti-inflammatory and immunomodulatory functions.

### Exploratory machine learning analysis of FMI and mortality

To capture the complex, non-linear associations between microbial taxa, clinical features, and frailty-related outcomes, we implemented XGBoost machine learning models to explore associations with FMI and all-cause mortality risk. The top 60 taxa associated with FMI identified in the full dataset were selected as candidate microbial features. These were combined with clinical features, including age, BMI, education level, smoking, alcohol intake, medication use and gene richness. These qualitative analyses enabled a data-driven prioritization of features, revealing distinct microbial signatures and clinical contributors to each outcome. Qualitative assessment of the relative feature importance analysis showed that the most influential taxa for FMI included *F. prausnitzii* clade I*, Faecalibacterium* sp900539945 and sp900539885, *Clostridium AP scindens*, *S. intestinalis, Massilioclostridium methylpentosum* and *Mediterraneibacter torques* (Supplementary Fig. 6). Notably, several of these microbial species showed comparable importance to microbiome gene richness and clinical predictors, such as BMI and medications (Supplementary Fig. 6). The Shapley Additive Explanation values (SHAP) were used solely to interpret the internal contribution of each feature to the XGBoost models. The mortality-specific XGBoost model (Supplementary Fig. 7) identified several taxa that were also associated with FMI (e.g., *Faecalibacterium* sp900539885 and sp900539945, *M. methylpentosum* and *M. torques*), alongside conventional predictors such as age, BMI, smoking and microbiome gene richness. These results suggest partially overlapping microbial features associated with FMI and mortality.

### Species-level concordance in the Chinese elderly cohort

**Fig. 6:**
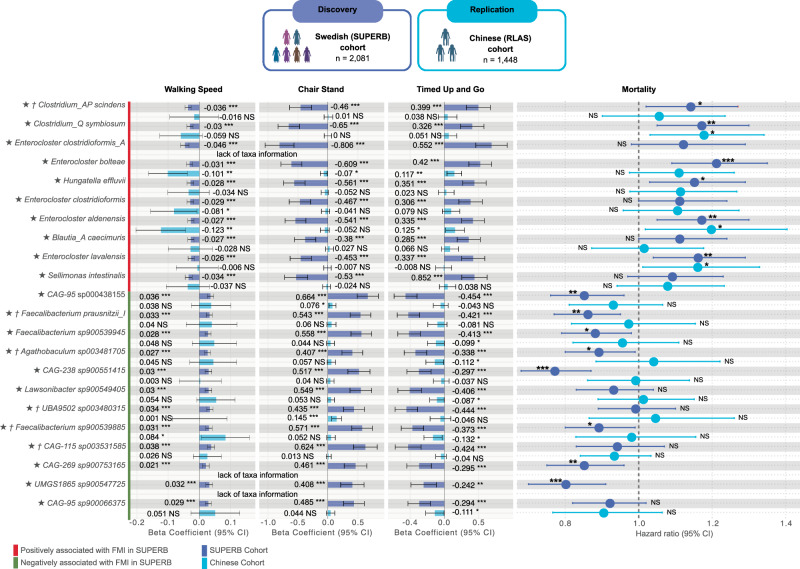
Species-level concordance of FMI-associated species in the SUPERB and Chinese elderly cohorts. Associations between bacterial species and clinical phenotypes, including walking speed, chair stand, timed up-and-go, and mortality, were evaluated in the SUPERB cohort (*n* = 2081) and examined in an independent Chinese elderly cohort (*n* = 1448). Of the top 5% FMI-associated species from SUPERB, 11 showed concordant species-level associations in the Chinese cohort, with consistent associations with physical function and/or mortality. In the SUPERB cohort, walking speed was measured in meters per second and chair stand performance as the number of repetitions completed in 30 seconds; higher values indicate better physical function. In the Chinese cohort, both measures were recorded in seconds, where higher values indicate poorer performance. To facilitate visual comparison, the β coefficients for these two measures in the Chinese cohort were directionally inverted for visualization only. Timed up-and-go was measured in seconds in both cohorts and was not transformed, as higher values consistently indicate poorer performance. Original β coefficients are reported in Supplementary Data 13. Beta coefficients from linear regression models are unstandardized, and species prevalence is shown as the percentage of participants carrying each taxon. Cox proportional hazards models were used to estimate hazard ratios for clinical outcomes. Colored bars indicate adjusted beta coefficients, points represent hazard ratios, and error bars denote 95% confidence intervals. Effect sizes, confidence intervals, and *P* values are provided in Supplementary Data 13. *P* values were two-tailed and were not adjusted for multiple comparisons. CI confidence interval, FMI Frailty Mortality Index, HR hazard ratio, RLAS Rugao Longitudinal Ageing Study; ***P ≤ 0.001; **0.001 < *P* ≤ 0.01; *P < 0.05; NS, not significant; ★ Species that remained significantly associated with FMI when adjusting for comorbidities; † species that remained statistically significant after adjustment for gene richness. Source data are provided as a Source Data file. Some elements in this figure were created in BioRender. Vilar Geraldi, M. (2026) https://BioRender.com/cre8nv5. The final figure layout was assembled in Affinity Designer 2.

To validate the utility of FMI and its associations with gut microbiota species, we sought to replicate our findings in an independent cohort. Species associated with FMI in the SUPERB cohort were tested for association with physical function parameters and mortality in the Chinese elderly cohort^18^. Among the top 5% species most strongly associated with FMI, 11 species (55%) showed significant associations in the same direction in the Chinese cohort. Overall, 52.3% of all FMI-associated species showed concordant associations with at least one physical function measure and/or mortality (Fig. 6 and Supplementary Data 13). Species showing significant negative associations with physical function in both cohorts were *Enterocloster* spp. (i.e. *E. bolteae, E. aldenensis*, and *E. clostridioformis)*, whilst species with positive associations were all uncharacterized and included *Faecalibacterium* sp900539945 and sp900539885, *Agathobaculum* sp003481705, and *Lawsonibacter* sp900549405 together with other MAGs with unknown taxonomy also at the genus level (e.g., CAG95 sp000438155, and UBA9502 sp003480315) (Fig. 6). *E. aldenensis* together with *E. lavalensis* and *Clostridium Q symbiosum*, were also significantly associated with mortality in both cohorts (Fig. 6 and Supplementary Data 13).

## Discussion

Frailty is a multidimensional syndrome of aging that is not fully captured by traditional comorbidity-based indices. To better identify gut microbiome species associated with relevant frailty-related mortality risk in the SUPERB cohort, we developed and internally validated the Frailty Mortality Index (FMI), a composite measure derived from clinical, functional, and lifestyle parameters that reflect physiological reserve, physical performance, and vulnerability to adverse outcomes. While the FMI conceptually overlaps with established frailty frameworks such as the Rockwood Frailty Index^6^ and Fried Phenotype^1^, it differs in scope and purpose: rather than serving as a clinical assessment tool, the FMI integrates age, smoking status, body weight, comorbidity, performance-based measures (e.g., walking speed, chair stand), mental health, and prior hospitalization into a single, weighted index derived from mortality associations. The FMI showed stronger associations to mortality, hip fracture, and fall-related injuries compared to the CCI, and explained a greater proportion of gut microbiota variation (0.6% vs. 0.2%), indicating that it may capture additional physiological dimensions relevant to host–microbiome interactions. The FMI was strongly associated with microbial diversity, gene richness, and altered taxonomic and functional composition. Our analyses identified both established clinical predictors of frailty and mortality as well as gut microbial species-level features that were independently associated with these outcomes and were not merely reflections of comorbid disease burden. Several of the most strongly associated species showed concordant patterns in an independent cohort of older Chinese adults, supporting species-level associations to aging, functional decline, and survival.

The decline in species diversity and reduced functional gene richness with increasing frailty severity aligns with previous research indicating that reduced microbial diversity is a hallmark of dysbiosis and degrading health in aging populations^18,20^. This reduction likely reflects a disruption of the ecological balance of the gut microbiota, leading to reduced resilience against pathogens and impaired metabolic capacity. Prior studies have reported that healthy aging is characterized by a gradual loss of core taxa and a greater microbial uniqueness^12,21,22^, whereas frailty is related to a depletion of key commensal species and an enrichment of pro-inflammatory, opportunistic, and facultative anaerobic bacteria^23,24^. In our study, lower gene richness, a proxy for microbial functional potential, was significantly associated with increased mortality and injurious falls. By incorporating gene richness as a covariate in a sensitivity analysis, we identified taxa specifically associated with frailty, beyond general microbial decline, that have not been considered in prior research.

Among the strongest microbial predictors of frailty and mortality in the SUPERB cohort, even after adjusting for gene richness, were *Enterocloster* species, known opportunistic pathogens, and *S. mutans*, a facultative anaerobe found in the oral cavity. The presence of oral taxa in fecal samples has previously been linked to depletion of commensal gut bacteria and adverse patient outcomes^25^. Their persistence despite controlling for gene richness suggests that their enrichment may reflect frailty-related processes such as barrier dysfunction or inflammation captured by the FMI. The abundance of *Clostridium Q symbiosum* was associated with frailty in the SUPERB cohort and with mortality both in SUPERB and in the Chinese elderly cohort, and has also been linked to unhealthy aging in previous studies^22,26^. *Enterocloster* species *E. bolteae*, *E. aldenensis*, and *E. lavalensis* were also associated with adverse outcomes and confirmed in the Chinese elderly replication cohort, and in previous studies^22,26^. Besides that, other FMI-associated species like *E. citroniae*, *E. clostridioformis*, *E. asparagiformis*, *C. innocuum*, *E. lenta, Ruminococcus gnavus* and *Flavonifractor plautii* were also previously identified as unhealthy aging markers^22,26^. These species are known to cause infections when the gut barrier is compromised and have been linked to antibiotic resistance^27^. Additionally, taxa associated with FMI were related to predicted butyrate production capacity. As butyrate promotes colonization resistance, supports regulatory T cell function, and dampens inflammation^28^, its reduction may weaken host defences and allow expansion of these opportunistic species, suggesting associations between predicted microbial metabolic capacity and frailty-related phenotypes.

The analyses of gut microbiota metabolic potential were consistent with the species-level profiles associated with FMI. GMMs enriched in individuals with severe frailty included pathways related to amino acid degradation, lactate metabolism and propionate production, trimethylamine/trimethylamine-N-oxide metabolism, and anaerobic respiration, consistent with the enrichment of oral, opportunistic and facultative anaerobic taxa among the species positively associated with FMI, and compatible with a shift toward protein fermentation and stress-adapted metabolism. Several of these predicted functions were linked to adverse clinical outcomes, including mortality and injurious falls, suggesting that microbiome metabolic functions may reflect frailty-related vulnerability. GMMs depleted with higher FMI captured predicted functions linked to gut and metabolic health, including methanogenesis from carbon dioxide, triacylglycerol degradation, pectin degradation, cysteine biosynthesis/homocysteine degradation, as well as acetate and butyrate production. Quantification of terminal genes for butyrate production showed a stepwise decline in the *but* gene and an increase in protein-derived butyrate pathways (*ato*A/D) in severely frail individuals, consistent with the increased amino acid metabolism revealed by GMM profiles. *F. prausnitzii* and uncharacterized *Faecalibacterium* MAGs were among the strongest taxa negatively associated with FMI and were also associated with better physical function in the Chinese elderly cohort. These results are consistent with previous findings^18,23^ and with the established role of *F. prausnitzii* as a dominant *but*-containing butyrate producer in the human gut^29,30^. Similarly, the abundance of *Dysosmobacter* sp900542115 was also associated with butyrate production potential and lower FMI severity. *Dysosmobacter welbionis*, the type species of the genus^31^, is a butyrate producer negatively associated with BMI, fasting glucose and glycated hemoglobin in overweight individuals and protective against obesity, insulin resistance and adipose tissue inflammation in mice^32^. Whilst these results support a role for butyrate-producing bacteria, butyrate production potential was not significantly associated with frailty-related clinical outcomes. Anti-inflammatory properties of *F. prausnitzii* and other gut bacteria may be particularly relevant^29,30^, especially given the increase in systemic inflammation (as measured by SIRI) observed with higher FMI scores. However, our functional profiling of the metagenomes was limited to metabolic capacity, and future studies are needed to clarify the role of bacterial anti-inflammatory activities in frailty and aging.

We also identified taxa uniquely associated with FMI after adjusting for gene richness, such as *Dysosmobacter* sp900770295 and *Massilistercora* sp902406105, which may have been overlooked in previous analyses not accounting for generalized microbiome depletion^33^. While the functional roles of these taxa remain unknown, they may represent microbes favored in conditions of altered microbiota composition^10,24^. Together with the identification of known and uncharacterized taxa negatively associated with frailty and mortality, these findings reinforce known patterns of frailty-associated dysbiosis, marked by loss of commensals and enrichment of inflammation-related taxa, and highlight candidates for future mechanistic and therapeutic investigations to delay or mitigate functional decline in older adults^20^.

Our findings are consistent with recent research showing that aging and frailty are accompanied by gut microbiome dysbiosis, characterized by loss of beneficial, SCFA-producing commensals and enrichment of pro-inflammatory and opportunistic bacteria^20,22,26^. Pu et al.^18^ reported reduced diversity and significant microbial shifts in frail individuals, identifying 162 species associated with frailty. Our results align with evidence that gut microbiota composition influences systemic inflammation, physical and cognitive function, which are key components of frailty^34^. Many of the taxa linked to FMI in our cohort have been consistently associated with frailty and unhealthy aging across multiple independent studies^18,22,26^ and showed concordant associations in the Chinese cohort, supporting the robustness of our findings across continents. Moreover, our analyses extend previous work by evaluating these associations independent of comorbidities, medication use, and gene richness.

Despite the strengths of our study, several limitations should be acknowledged. The cross-sectional nature of the analysis precludes causal inference, making it difficult to determine whether gut microbial changes precede frailty onset or are a consequence of aging-related physiological decline. Furthermore, owing to data limitations in the SUPERB cohort, we were unable to compare the FMI with the Fried Frailty Phenotype^1^ or the Rockwood Frailty Index^6^. Bacteriophage and archaeal communities were not assessed, preventing evaluation of their potential contribution to frailty-related dysbiosis. Metabolic inferences were derived from microbial gene module profiles rather than direct metabolomic measurements. As such, the predicted alterations in SCFA, amino-acid, and lipid metabolism reflect potential functional capacity rather than measured metabolite concentrations. Our analyses were conducted at the species level and do not capture strain-level heterogeneity, which may be particularly relevant across geographically distinct cohorts. As strains within the same species can differ in gene content and function, the associations reported in this study should not be interpreted as reflecting shared functional lineages across populations. Although we observed consistent species-level associations in the independently analyzed Chinese cohort using a harmonized bioinformatic framework, mechanistic interpretation remains limited without strain-resolved analyses, which will be important to address in future studies. Another limitation is the low statistical power regarding the mortality outcome (*n* = 99) in the replication cohort, which likely reduced the number of species significantly replicated. The discovery cohort consisted exclusively of older women from Sweden, but most of the top-ranking species were consistently associated with physical function and/or mortality in the Chinese elderly cohort, including both men and women (women, 54%) with ages ranging from 62–96 years. An additional limitation is the lack of data regarding dietary intake, which is known to affect the gut microbiota. Future longitudinal studies incorporating more diverse cohorts and external replication datasets are necessary to validate these findings and assess the broader applicability of microbiome-based frailty biomarkers.

In conclusion, the FMI integrates weighted clinical, functional, and psychosocial components associated with mortality risk in the SUPERB cohort. Using the FMI, we identified gut microbiota species-level features associated with FMI severity, frailty-related adverse clinical outcomes, and mortality. Translation into therapies will require further strain-level research, isolation of specific bacterial taxa, and identification of microbial functions relevant for interaction with the host and involved in frailty-related processes.

## Methods

This study was approved by the Regional Ethics Review Board of Gothenburg and followed the ethical standards of the Declaration of Helsinki 1964 and its later amendments. The RLAS cohort study was approved by the Ethics Committee of the Fudan University School of Life Sciences (No. BE1815). All participants signed informed consent forms, and no compensation was offered to participants.

### Study design and subjects

The current study was based on the Sahlgrenska University Hospital Prospective Evaluation of Risk of Bone Fractures (SUPERB) study, a population-based longitudinal study conducted in the Gothenburg area, Sweden, between 2013 and 2016^35^. The study cohort included 3028 women aged 75–80 years who were randomly selected using the Swedish National Population Register (NPR). The participants’ legal sex was determined from the NPR. Because the study population consisted exclusively of female participants, no sex-stratified analyses were performed. Eligibility criteria for participation in the study required that the participants were from the Swedish population, had sufficient understanding of Swedish, were able to attend scheduled clinical visits, and were ambulatory. Study participants were followed from inclusion to time of death or study end in March 2023. A schematic diagram showing the study design and methodological procedures for SUPERB is provided in Fig. 1.

### Anthropometrics, mortality, injurious falls and hip fracture assessments

Anthropometric measurements were conducted using standardized protocols at baseline. Body height and weight were quantified utilizing a uniform wall-mounted calibrated stadiometer and scale, respectively, for all participants. The mean values of height and weight were derived from two consecutive measurements. BMI was calculated as weight (kg) divided by height squared (m^2^). Dual-energy X-ray absorptiometry (DXA; Hologic Discovery A, Hologic, Waltham, MA, USA) was used to assess lean mass, and the Appendicular Lean Mass Index (ALMi) was calculated by dividing the total lean mass of the arms and legs (kg) by height squared (m^2^).

Mortality during follow-up was retrieved from the regional population registry Västfolket. Hip fractures were verified through a comprehensive review of radiology reports and/or images obtained from the Regional Digital X-ray Archive, which encompasses 49 municipalities in the Västra Götaland Region surrounding Gothenburg, Sweden. Research nurses screened all radiology reports from baseline until March 2023, and cases with missing reports or uncertain diagnoses were reviewed by an orthopedic surgeon. Data on incident injurious falls were retrieved from the National Patient Registry maintained by the National Board of Health and Welfare (Socialstyrelsen). Participants were followed from baseline until the first occurrence of hip fracture, death, or the end of follow-up (March 2023). Follow-up for injurious falls extended until December 31, 2021, corresponding to the latest National Patient Register extract.

### Baseline data collection – questionnaires and registry data

The questionnaire protocol was structured into two distinct components: a self-administered form and a supplementary form completed in conjunction with a research nurse during the inclusion visit. These instruments were designed to capture a comprehensive array of clinical risk factors, including current smoking status^36^, medical and fracture history, incidence of accidental falls within the preceding 12-month period, parental history of hip fracture, alcohol consumption patterns, and the highest achieved education level. Alcohol consumption was quantified using the AUDIT questionnaire^37^, facilitating the derivation of a variable indicative of excessive consumption (defined as exceeding 21 standard drinks per week). The Short Form survey-12 (SF-12) questionnaire was employed to gather data pertaining to physical and self-reported quality of life, yielding both physical (PCS) and mental (MCS) component scores^38^. Additionally, detailed information regarding physical activity habits was collected through the administration of the Physical Activity Scale for the Elderly (PASE) survey^39^.

Data on prescribed medications (antibiotics, statins, metformin and proton pump inhibitors) were collected from the National Prescribed Drug Register (The National Board of Health and Welfare). Current use was defined as having 2 prescriptions in the last year, of which at least one was in the past 120 days.

### Physical function tests

The physical function assessment protocol encompassed five distinct tests, administered at baseline under the supervision of research personnel: hand grip strength, timed up and go (TUG, seconds), one-leg standing (OLS; seconds), walking speed (meter/second), and chair stand test (number of raises in 30 seconds). Hand grip strength was quantified using a Saehan hydraulic hand dynamometer (model SH5001; Saehan Corporation, Masan, Korea), with the dominant hand evaluated twice while the arm rested on a table at a 90° elbow flexion; the mean of these measurements (in kilograms) was utilized for analysis^40^. The TUG test evaluated mobility and balance, measuring the time required for participants to rise from a 45 cm high chair with armrests, walk 3 meters at a normal pace, turn around, return, and sit down, with the use of regular footwear and mobility aids permitted as needed^41–43^. The OLS test, a clinical balance assessment, was performed with eyes open, barefoot, arms crossed over the chest, and standing on one leg with the other flexed posteriorly at the knee; following a practice session, two trials per leg were conducted, with a maximum duration of 30 seconds, and the highest value was employed in the analysis^44,45^. Gait speed was assessed via the timed 10-meter walk test, wherein participants traversed 10 meters at a comfortable pace, with timing initiated at the 2-meter mark and concluded at the 8-meter point to account for acceleration and deceleration; the mean of two trials, recorded in meters per second, was utilized^46,47^. Lower body strength was evaluated using the 30-second chair stand test, which required participants to repeatedly stand up from and sit down on a 45 cm high chair, with arms crossed over the chest, as many times as possible within a 30-second interval^48^.

### Frailty Mortality Index and the Charlson Comorbidity Index

The Frailty Mortality Index (FMI) is a composite measure designed to quantify frailty-associated mortality risk by integrating multiple parameters. FMI was developed in the full SUPERB cohort of community-dwelling women aged 75–80 years at baseline (*n* = 3028). The index was derived from 16 investigated frailty- and mortality-associated factors using Cox proportional hazard models with death as the outcome over 7.9 years of median follow-up. Predictors included comorbidity, physical function, anthropometrics and lifestyle parameters, resulting in a weighted model with remaining contributing factors, which included age (years), current smoking status (yes/no), mental quality of life (SF12), CCI, walking speed, chair stand test performance, body weight (kg), and hospital stay duration history (days). Details of the FMI, score weights, and their associations with clinical outcomes are presented in Fig. 1c and individual scores in the Figshare repository^49^. The FMI was internally validated using leave-one-out cross-validation (LOOCV), showing similar discrimination for mortality before and after validation (C-index 0.688 and 0.683). The cohort (*n* = 3028) was divided into groups according to quartiles of the FMI, that is, 1 = no, 2 = mild, 3 = moderate, and 4 = severe. The CCI^9^ was calculated based on information on diseases collected through baseline questionnaires and ICD-10 codes retrieved from the National Patient Register (The National Board of Health and Welfare).

### DNA extraction from fecal samples and whole genome metagenomic sequencing

Stool samples were collected at participants’ homes at baseline and stored in the fridge for a maximum of 36 hours before storage at −80 °C. Fecal genomic DNA was extracted from 100–150 mg of fecal material using repeated bead beating in Lysing Matrix E tubes (MP Biomedicals) containing lysis buffer (4% w/v SDS; 500 mmol/L NaCl; 50 mmol/L EDTA; 50 mmol/L Tris·HCl; pH 8)^50^. Two bead beating cycles were performed at 5.5 m/s for 60 s in a FastPrep®−24 Instrument (MP Biomedicals), and before each cycle samples were heated at 95 °C for 5 min. Samples were placed on ice for 5 min in between the two bead-beating cycles. After each bead-beating cycle, samples were centrifuged at full speed for 5 min at 4 °C, and supernatants from the two cycles were pooled. A 600 µL aliquot from each sample was purified using the QIAamp DNA Mini kit (QIAGEN) in a QIAcube (QIAGEN) instrument using the procedure for human DNA analysis. Samples were eluted in 200 µL of AE buffer (10 mmol/L Tris·Cl; 0.5 mmol/L EDTA; pH 9.0). Libraries for whole-genome metagenomic sequencing were prepared by a PCR-free method; library preparation and sequencing were performed at Novogene (UK) on a NovaSeq instrument (Illumina) with 150-bp paired-end reads and 7.5 GB data per sample.

### Metagenome analyses

Illumina reads were quality filtered and trimmed using fastq_quality_trimmer from the FASTX Toolkit (https://www.bioinformatics.babraham.ac.uk/projects/fastqc/); human reads were removed by mapping the high-quality reads against the human genome (hg19) using Bowtie2^51^ (v2.4.4). After removal of low-quality (quality score <20) and human reads, we obtained high-quality paired-end microbial reads with an average depth of 36 million for each sample. Batch corrections were performed using metadeconfoundR (https://github.com/TillBirkner/metadeconfoundR). Relative abundances of species were obtained by mapping high-quality reads to the Unified Human Gastrointestinal Genome (UHGG) version 2.0 catalog (https://www.ebi.ac.uk/metagenomics/genome-catalogues/human-gut-v2-0)^33^ using Kraken2 version 2.1.2^52^, and abundance profiles were generated using Bracken version 2.6.2^53^.

Gene counts in the metagenomic data were estimated using MEDUSA^54^ with a gene catalog containing 15,186,403 non-redundant microbial genes^50^. Microbial genes for butyrate potential production were quantified based on five genes (*but*, *buk*, *4hbt*, and *ato*D) coding for the terminal enzymes in the main pathways of human intestinal butyrate-producing^55^. Profile hidden Markov models were used to screen those genes in the gene catalog. Gene counts were rarefied to 36 million reads per sample. Gene richness in each sample was calculated based on the number of genes present from the 36 million rarefied reads gene counts. Differential abundance was calculated using Deseq2^56^. For metagenome functional analysis, genes were annotated using the KEGG Orthology (KOs) database and gene read counts were collated into functional gene families. Tests for differential abundance of KO functions were performed using ANCOM-BC. KOs were summarized into gut metabolic module (GMM) profiles, using Omixer-RPM^57^. The functions utilized in these analyses are implemented in the vegan package (Community Ecology Package-R package version 1.17-8). All statistical analyses involving fecal whole-genome metagenomics were performed in R (version 4.1.0).

### Alpha-diversity, gene richness, beta-diversity and PERMANOVA analysis

The beta-diversity of gut microbiome between samples was calculated using species-level Aitchison distance (Euclidean distance of inverse rank-transformed abundance data) and visualized using principal coordinate analyses (PCoA) plots. We applied permutational multivariate analysis of variance (PERMANOVA) with 999 permutations to quantify the percentage of variance in the relative abundance of microbial species explained by FMI groups and covariates based on the Aitchison dissimilarity metric using the ‘adonis2’ function in the R package ‘vegan’ (version 2.6-6.1). A sensitivity analysis using Bray-Curtis dissimilarity was performed with ordination by PCoA and the same PERMANOVA framework. Gut microbiome alpha-diversity was obtained as the Shannon index calculated using a species-level relative abundance matrix. Gene richness was defined as the total unique gene count in each sample.

### Replication cohort

To validate the associations between microbial species and the FMI, we conducted parallel analyses using same bioinformatics pipeline in an independent population of older Chinese adults, recruited from Rugao, Jiangsu Province, China (Rugao Longitudinal Ageing Study, RLAS). This replication cohort included 1448 participants (775 women and 673 men) with available gut metagenomic sequencing data, along with assessments of physical function^18^. To ensure harmonization, both cohorts were processed using identical bioinformatics workflows, including read trimming, host filtering, mapping against the UHGG reference catalog, and taxonomic profiling with the same quality-control thresholds. As the Chinese cohort lacked data required to calculate the FMI (hospital stay duration, mental QoL, and CCI), FMI-associated species identified in the SUPERB cohort were instead tested for association with physical function parameters and mortality. Physical function was assessed in the Chinese cohort using three performance tests: walking speed, chair rise, and the TUG test. Walking speed was measured as the time (in seconds) taken to walk 5 meters at a usual pace. Chair rise time was defined as the time (in seconds) required to stand up from a seated position once. The TUG test measured the time (in seconds) taken to rise from a chair, walk a short distance, turn around, return, and sit down again. For all measures, higher values indicate slower performance and thus poorer physical function. Cox proportional hazards regression was used for mortality, and linear regression was used for physical function tests, adjusting for age, education level, smoking status (never, former, or current), drinking status (never, former, or current), and medication use. Differences in measurement units for physical function outcomes were harmonized by inverting β coefficients where necessary to ensure consistent directionality of effects between cohorts, and these adjustments were clearly indicated in the corresponding figure and table legends.

### Statistics & Reproducibility

This study was a prospective cohort study and participants were followed longitudinally for frailty and mortality outcomes. No statistical method was used to predetermine sample size. Participants were not randomized, and no investigator blinding was conducted. Study participants with any prescription for oral antibiotics in the past 90 days (*n* = 276) or missing any of the parameters used to calculate the FMI (*n* = 41) were excluded from analysis, yielding a complete dataset of 2,081 study subjects. Only annotated bacterial reads with an absolute read count above 100 in at least 10% of the population were included in the analyses.

Associations between bacterial species and FMI were investigated using linear regression models, with FMI as the dependent variable and microbial species as independent predictors. The main model (*n* = 2081) was adjusted for age, BMI, smoking, alcohol intake, education level, use of statins, proton pump inhibitors, and metformin. A gene richness-adjusted model included the same covariates with additional adjustment for gene richness. Multiple-comparisons correction was performed using the Bonferroni method, accounting for the number of analyzed species (*n* = 2057), yielding a significance threshold of *P* < 2.43 × 10^-5^.

To assess whether the associations between gut microbiota and FMI were independent of comorbidity and baseline disease status, additional sensitivity and subgroup analyses were performed. The comorbidity-adjusted model (*n* = 2081) included all covariates from the main model plus individual comorbidities present at baseline, including rheumatoid arthritis, dementia, ischemic heart disease, heart failure, cerebrovascular disease, chronic pulmonary disease, liver disease, diabetes mellitus, renal failure, hemiplegia/paraplegia, peptic ulcer disease, solid non-metastatic tumor, lymphoma/leukemia, hyperparathyroidism, hyperthyroidism, and malnutrition.

FMI-associated species that remained significant after Bonferroni correction were further analyzed in relation to frailty-related parameters using the main model. Cox regression models were used to estimate HR with 95% confidence intervals for death, hip fracture, and injurious falls. Results of frailty-related parameters were adjusted using the Benjamini-Hochberg false discovery rate (FDR) method, with a q value threshold of 0.05 to determine statistical significance.

Extreme Gradient Boosting (XGBoost), an ensemble machine learning technique based on decision trees, was used as an exploratory approach to qualitatively assess the relative contribution of baseline features to FMI and mortality. The top 60 taxa associated with FMI identified in the full dataset were selected as candidate microbial features. These taxa were then combined with clinical variables including age, BMI, education level, smoking, alcohol intake, medication use, and gene richness. The method builds a multivariable ensemble of prediction models that can capture complex non-linear relationships and interactions among variables. For FMI and mortality, the optimal hyperparameters were identified by performing grid search over combinations of learning rate and maximum tree depth. For mortality, we used XGBoost with a Cox proportional hazards objective to account for right-censored data. Hyperparameter tuning was conducted using cross-validated root mean squared error (RMSE) and Cox negative log-likelihood for FMI and mortality, respectively. Survival time and event status (death = 1, censored = 0) were used as outcome variables. Feature importance was assessed using gain-based feature importance metrics and SHAP values for easy interpretation of the machine learning model output. The SHAP values reflect the model-derived contribution of each feature to XGBoost predictions and should not be interpreted as independent associations or causal effects. The SHAP value in this analysis is the mean absolute individual feature-level impact on the model. The training set in our models consisted of a randomly selected subset of 80% of the study participants, and the testing set was composed of the remaining 20%. The model was based on data from the training set; the testing set was independent of the training process and was used only for performance evaluation after the model was established. We note that feature selection was performed prior to cross-validation, which may introduce a degree of information leakage; therefore, model performance and feature-importance estimates were interpreted qualitatively. All models were implemented in R using the XGBoost package (version 1.6.0.1 for FMI and 1.7.5 for mortality).

ANOVA with Bonferroni post hoc test was used to test differences between FMI groups, between baseline continuous and normally distributed variables, Kruskal-Wallis H independent test for continuous and not normally distributed variables, and χ^2^ for dichotomous variables.

### Reporting summary

Further information on research design is available in the Nature Portfolio Reporting Summary linked to this article.

## Supplementary information

Supplementary Information

Description of Additional Supplementary Files

Supplementary Data

Reporting Summary

Transparent Peer Review file

## Source data

Source Data

## Data Availability

The whole-metagenome sequencing data generated in this study have been deposited in the European Nucleotide Archive (ENA) database under study accession code PRJEB110772 with public access. De-identified individual participant data are deposited in Figshare^49^. Other clinical data collected from this study will be available under restricted access for sensitive personal data protection, ethical restrictions, the General Data Protection Regulation (GDPR), and the Swedish Public Access to Information and Secrecy Act (SFS 2009:400); access can be obtained by submitting a data access request to Jan Boren (jan.boren@wlab.gu.se), head of the Institute of Medicine, Sahlgrenska Academy, University of Gothenburg, Gothenburg, Sweden. The RLAS cohort sequence data are available in the National Center for National Omics Data Encyclopedia under accession number code OEP001391. Source data are provided with this paper.

## Code availability

Code for analyses and figures is provided at Zenodo via 10.5281/zenodo.20489791 (ref. ^58^).
