# Supplementary material for: Gut microbiota associates with frailty in older women
Source: Nat Commun. 2026 Jul 8;17:5925. doi: 10.1038/s41467-026-75176-5 (PMC13346421; doi:10.1038/s41467-026-75176-5)
Supplement: Supplementary file 1 — Supplementary Information [file 41467_2026_75176_MOESM1_ESM.pdf]

Supplementary Figure 1 | Distribution of clinical and functional variables across Frailty Mortality Index groups.

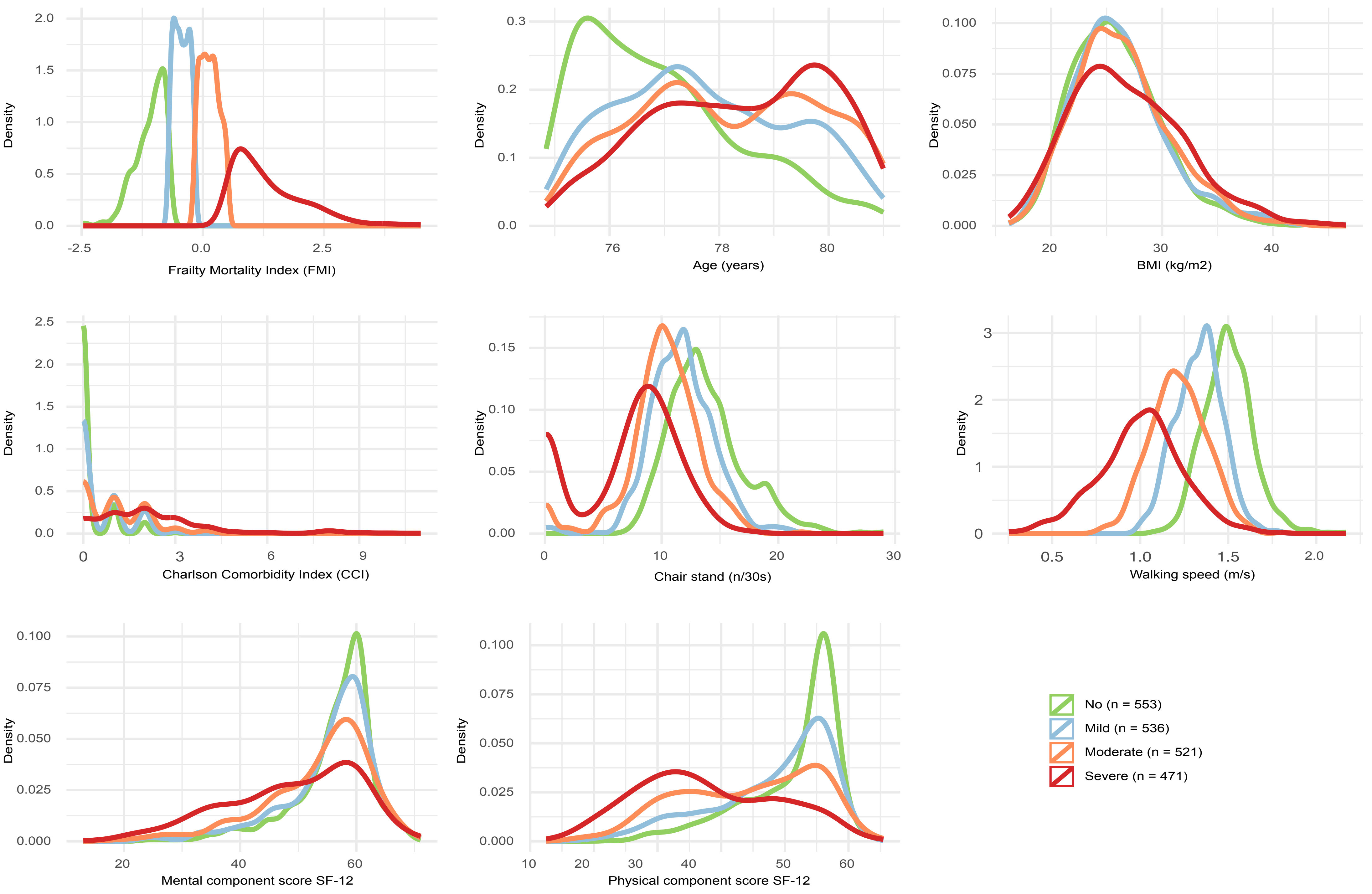

Supplementary Figure 1 | Distribution of clinical and functional variables across Frailty Mortality Index groups.

Density plots show the distribution of clinical and functional variables across groups, stratified by percentile-based categories of the Frailty Mortality Index (FMI). BMI, Body Mass Index; SF-12, 12-item Short Form Health Survey. N = 2,081. Source data are provided as a Source Data file.

Supplementary Figure 2 | Sensitivity analysis of gut microbiome composition using Bray–Curtis dissimilarity.

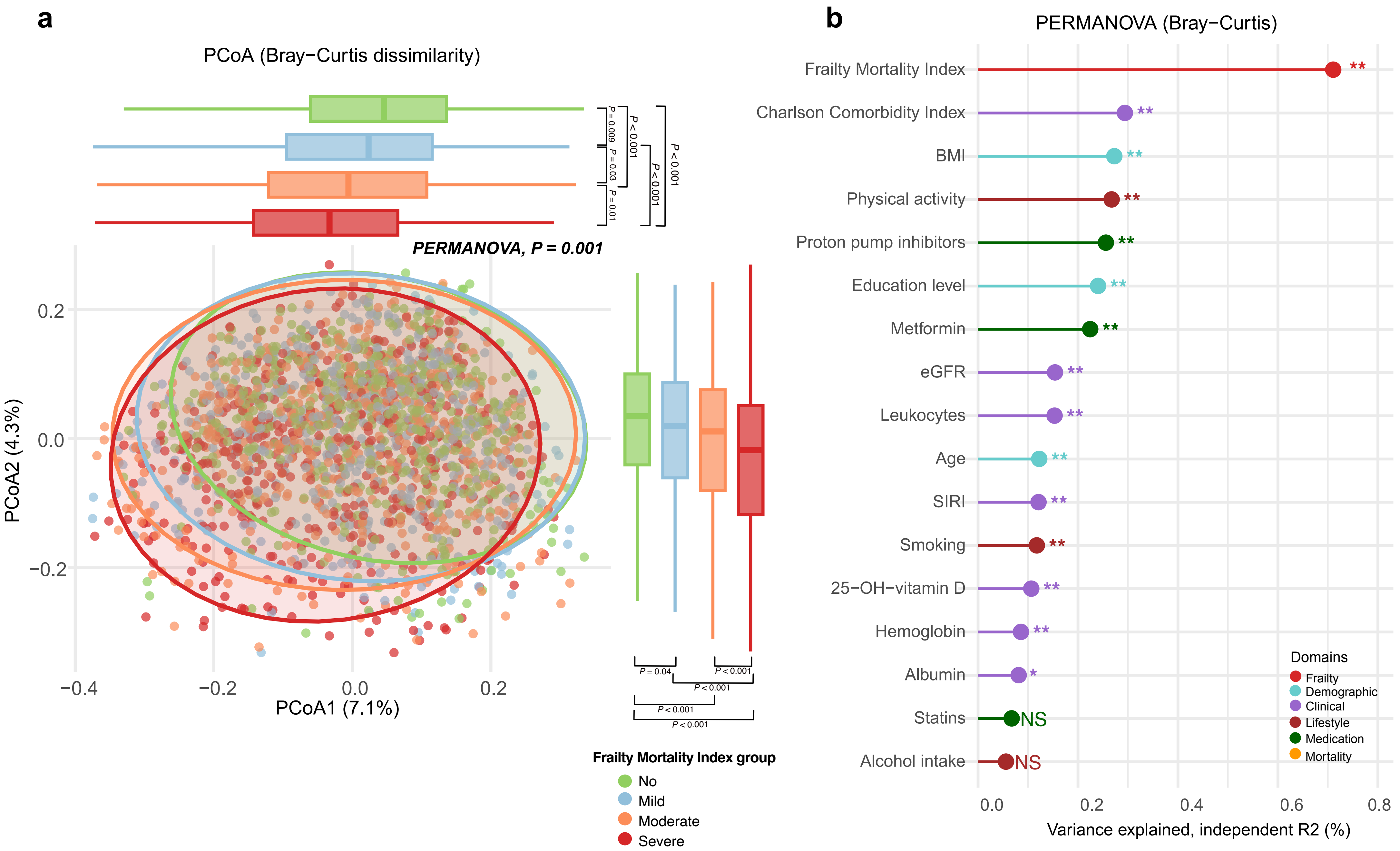

Supplementary Figure 2 | Sensitivity analysis of gut microbiome composition using Bray–Curtis dissimilarity.

a, Principal coordinate analysis (PCoA) based on Bray–Curtis dissimilarity showing distinct clustering of gut microbiota composition across FMI groups. Each point represents one participant, colored according to FMI category. Boxplots of the first two PCoA axes (PCoA1 and PCoA2) across frailty groups, showing significant differences in community structure between groups (Kruskal–Wallis; pairwise Wilcoxon tests with Benjamini–Hochberg correction).

b, Partial variance explained (PERMANOVA, 999 permutations) for frailty group and covariates, indicating that frailty (FMI group) remained significantly associated with gut microbiota composition.

N = 2,081. *** $P \leq 0.001$; ** $0.001 < P \leq 0.01$; * $P < 0.05$; NS, not significant. PCoA, principal coordinate; BMI, Body Mass Index; eGFR, estimated Glomerular Filtration Rate; SIRI, systemic inflammation response index; PERMANOVA, permutational multivariate analysis of variance. Source data are provided as a Source Data file.

Supplementary Figure 3 | Associations between Charlson Comorbidity Index and gut microbiome diversity.

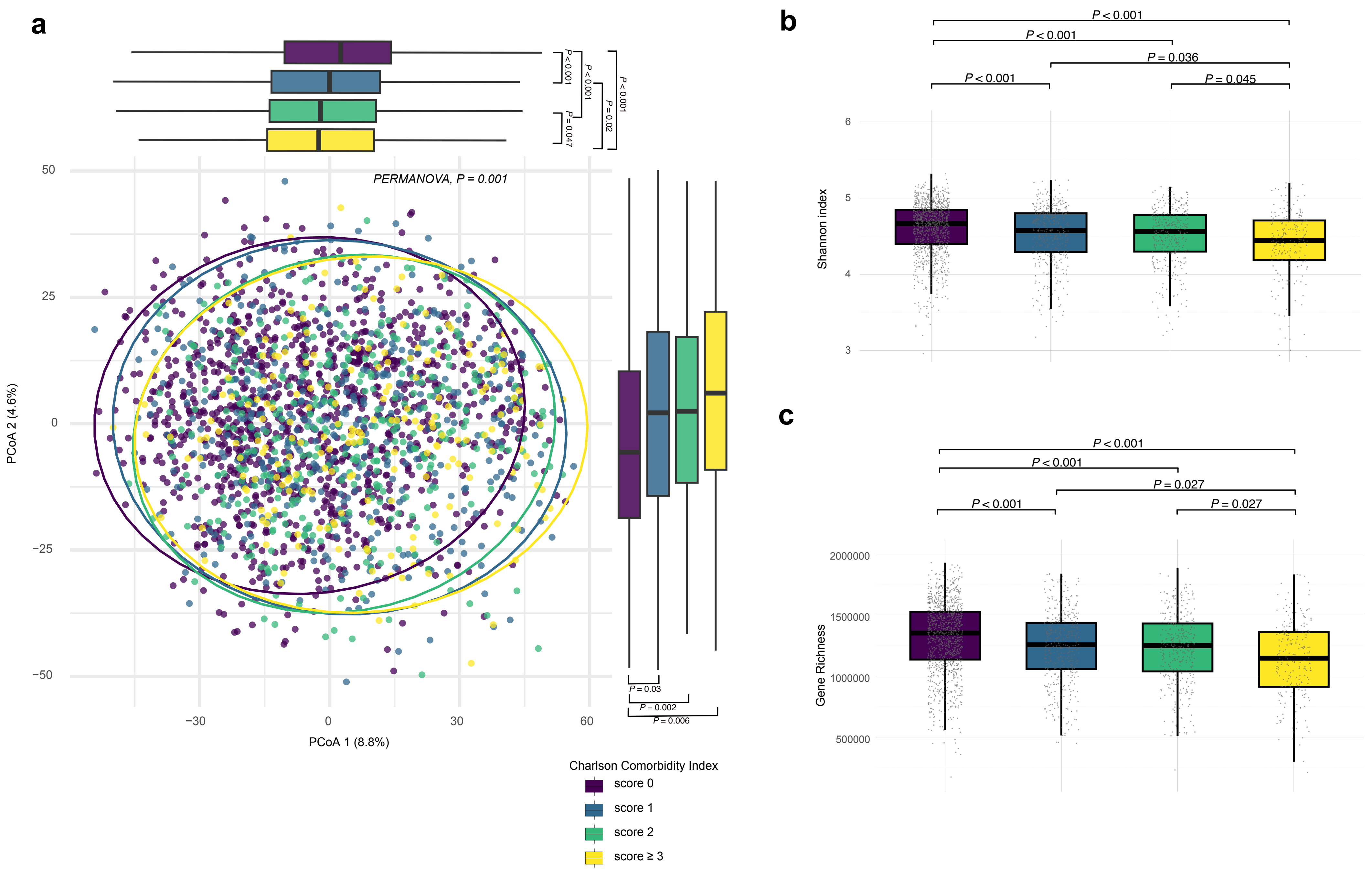

Supplementary Figure 3 | Associations between Charlson Comorbidity Index and gut microbiome diversity.

a, Principal coordinate analysis (PCoA) of species-level Aitchison distances reveals distinct clustering of gut microbial composition by Charlson Comorbidity Index group. Box plots along PCoA1 and PCoA2 axes show the distribution (median and interquartile range) of individual scores within each group.

b and **c,** Box plot illustrating the overall distribution of Shannon index (b) and gene richness (c) across frailty mortality groups.

P values were calculated using Kruskal-Wallis test and corrected for multiple comparisons using Bonferroni. These analyses are based on 2,081 participants. *** $P \leq 0.001$; ** $0.001 < P \leq 0.01$; * $P < 0.05$; NS. PCoA, principal coordinate; PERMANOVA, permutational multivariate analysis of variance. Source data are provided as a Source Data file.

Supplementary Figure 4 | Association of FMI-linked microbial species and clinical characteristics.

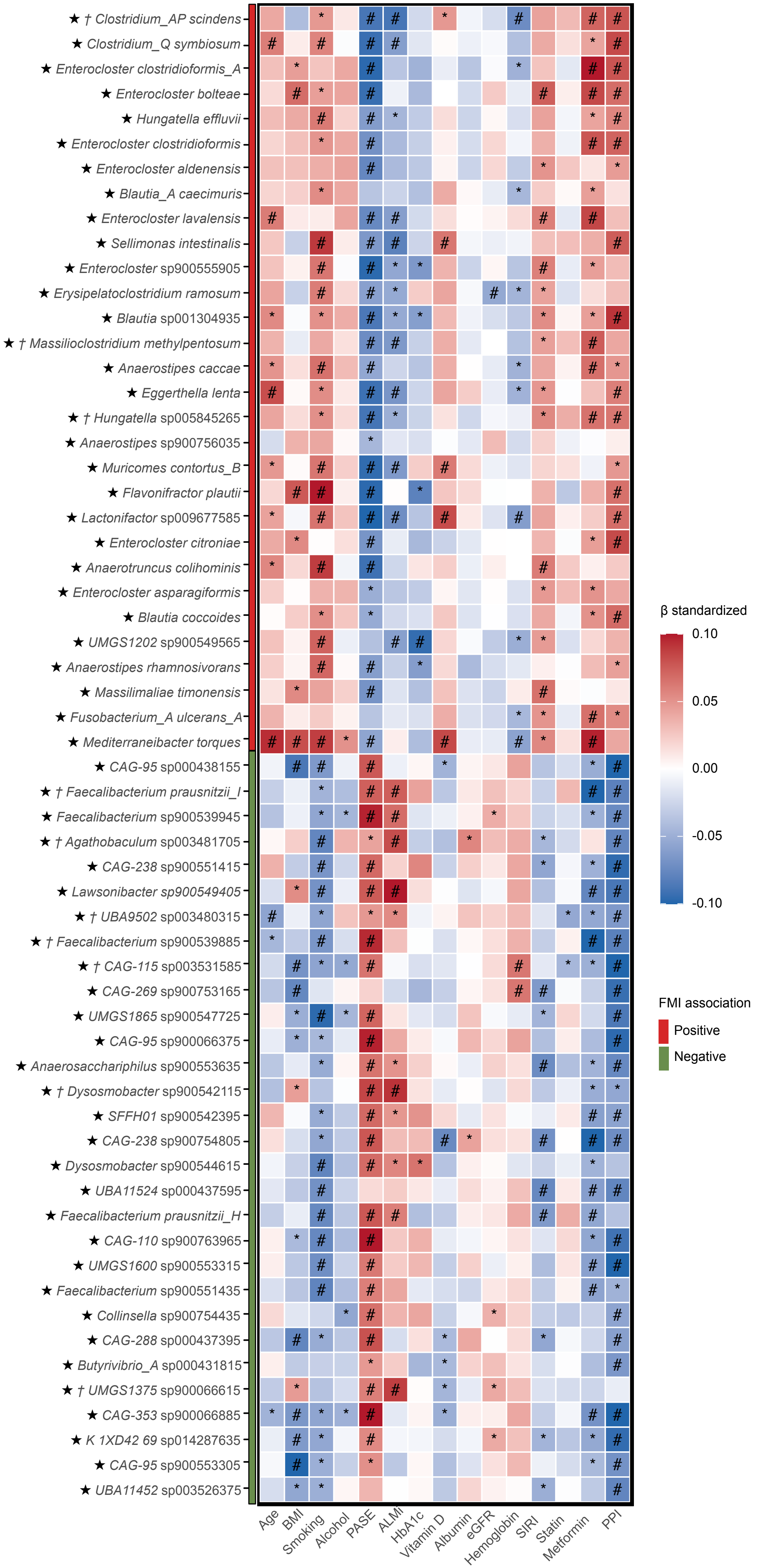

Supplementary Figure 4 | Association of FMI-linked microbial species and clinical characteristics.

Heatmap displaying standardized β coefficients from multivariable linear regression models evaluating the associations between the top 30 positive and 30 negative microbial species and clinical or biochemical variables. Microbial species were selected based on their significant association with the Frailty Mortality Index (FMI).

All models were adjusted for age, BMI, education level, smoking status, alcohol intake, statin use, proton pump inhibitor use, and metformin use, except when the respective variable was the outcome, in which case it was excluded from the adjustment. FMI, Frailty Mortality Index; BMI, body mass index; eGFR, estimated Glomerular Filtration Rate; PASE, Physical Activity Scale for the Elderly; ALMi, appendicular lean mass index; HbA1c, glycated hemoglobin; PPI, Proton pump inhibitors; SIRI, Systemic Inflammation Response Index. ★ Species that remained significantly associated with FMI after adjustment for comorbidities. # FDR ≤ 0.01; * FDR < 0.05. These analyses are based on 2,081 participants. Source data are provided as a Source Data file.

Supplementary Figure 5 | Microbial species positively and negatively associated with the Frailty Mortality Index (FMI) and butyrate production potential.

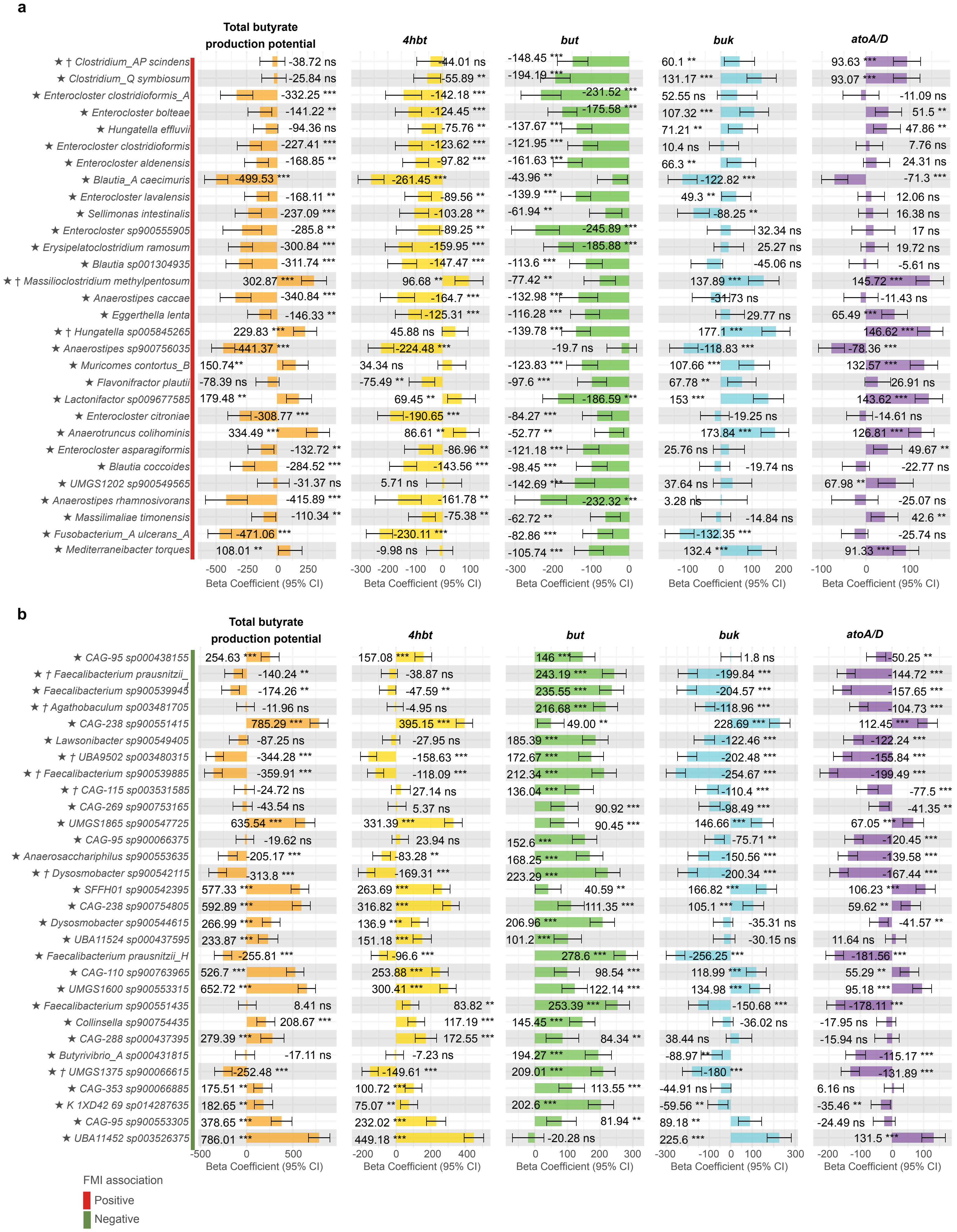

Supplementary Figure 5 | Microbial species positively and negatively associated with the Frailty Mortality Index (FMI) and butyrate production potential.

Associations between individual microbial species and butyrate production potential are shown. **a,** Species positively associated with FMI. **b,** Species negatively associated with FMI. β coefficients (95% CI) from linear regression models are unstandardized.

Analyses include 2,081 participants. All models were adjusted for age, BMI, education level, smoking status, alcohol intake, statin use, proton pump inhibitor use, and metformin use. ★ Species that remained significantly associated with FMI after adjusting for comorbidities; *** $P < 0.00012$ (Bonferroni-adjusted for 404 tests); ** FDR < 0.05; * FDR < 0.1; NS, not significant. CI, confidence interval. Source data are provided as a Source Data file.

Supplementary Figure 6 | Qualitative assessment of the relative importance of clinical and microbial features in predicting Frailty Mortality Index (FMI).

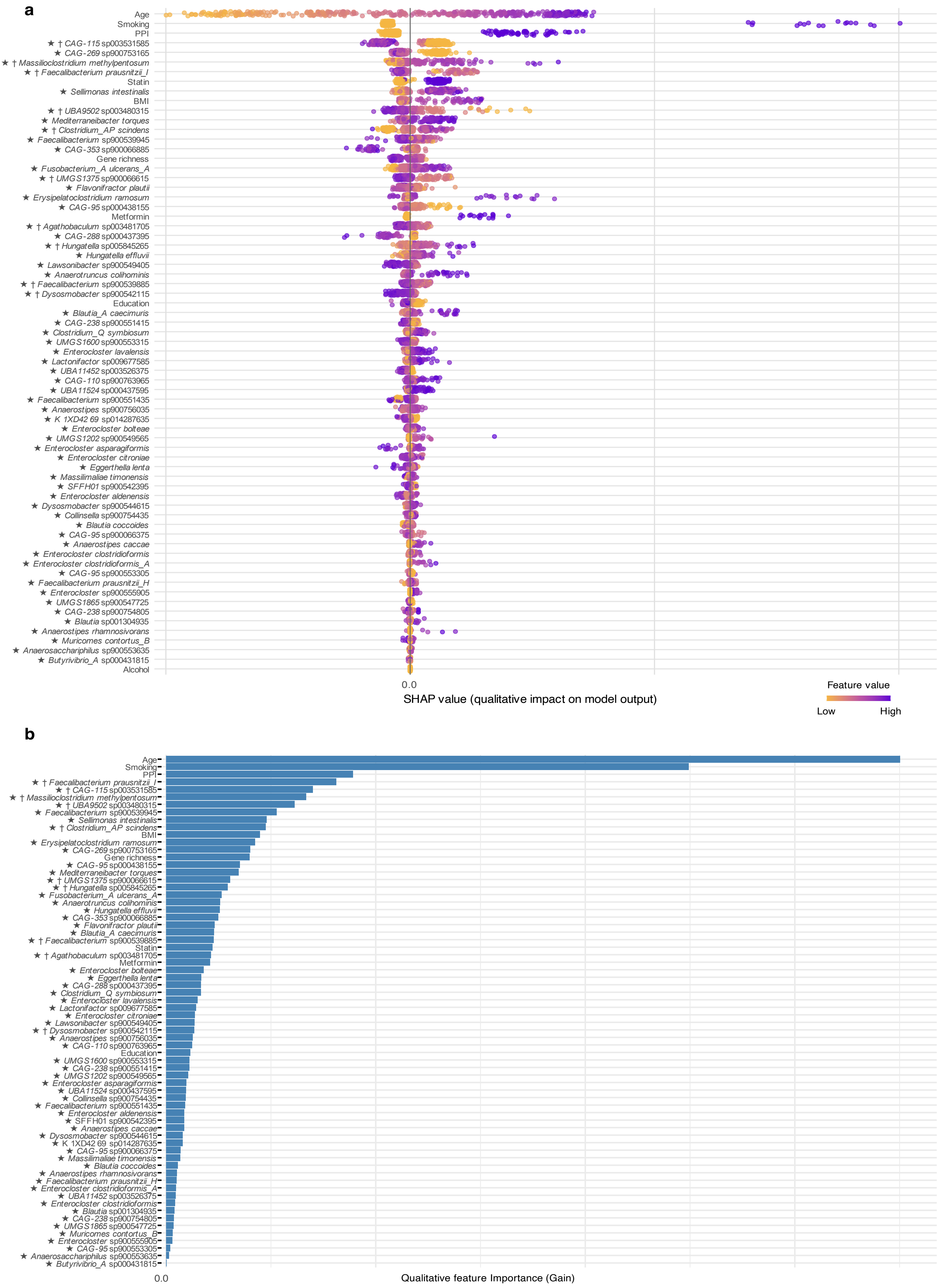

Supplementary Figure 6 | Qualitative assessment of the relative importance of clinical and microbial features in predicting Frailty Mortality Index (FMI).

a, SHAP summary plot showing the qualitative contribution of individual clinical and microbial species-level features to the prediction of FMI. Each point represents one participant and indicates the magnitude and direction of each feature's impact on model output.

b, Qualitative assessment of the relative importance of features in predicting FMI, as estimated by an XGBoost model.

These analyses were based on 2,081 participants. † Species that remained statistically significant after adjustment for gene richness. PPI, proton pump inhibitor; BMI, Body Mass Index; SHAP, Shapley Additive Explanation Values. Source data are provided as a Source Data file.

Supplementary Figure 7 | Qualitative assessment of the relative importance of clinical and microbiota features in predicting mortality.

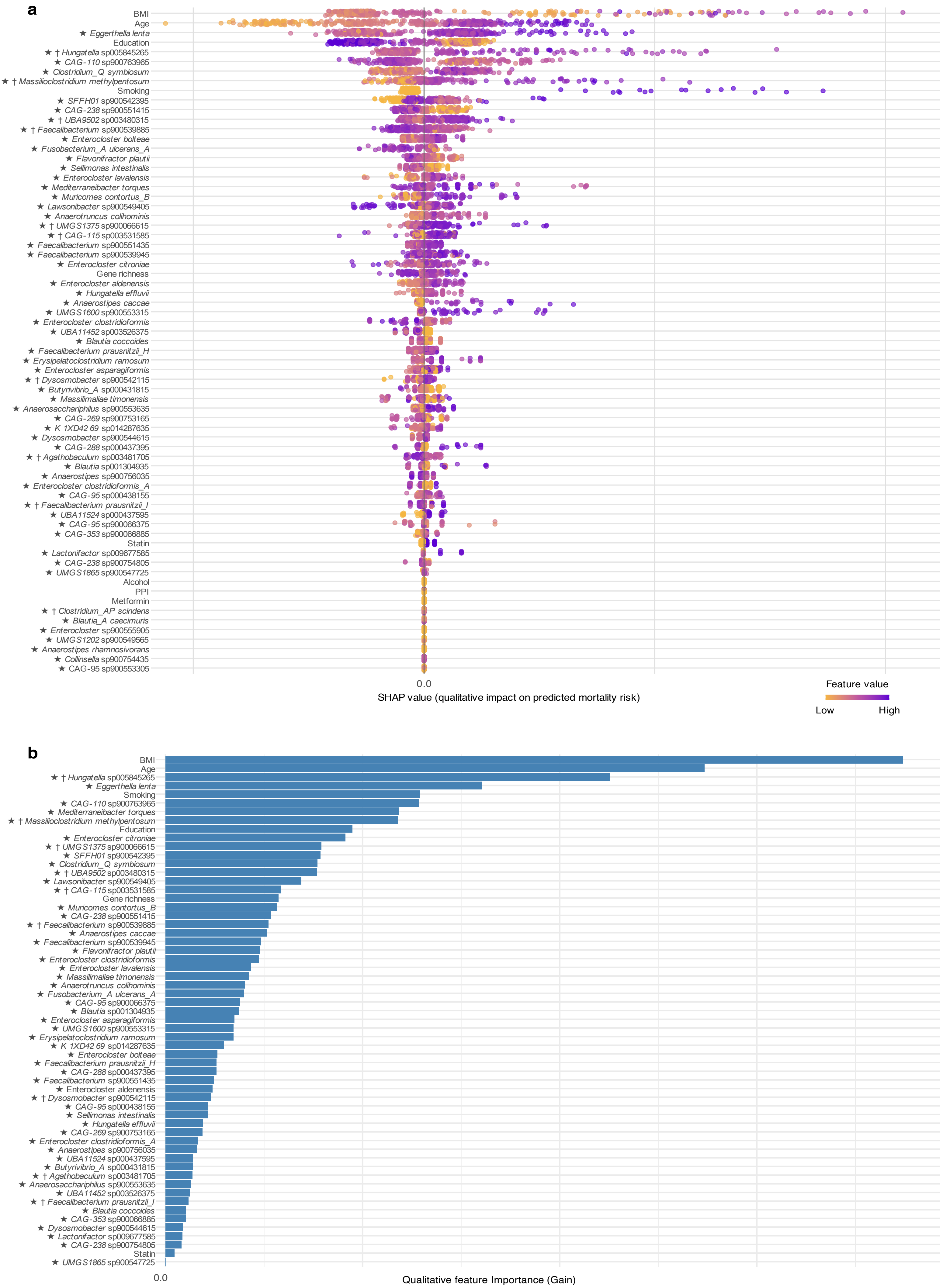

Supplementary Figure 7 | Qualitative assessment of the relative importance of clinical and microbiota features in predicting mortality.

a, SHAP summary plot showing the qualitative contribution of individual clinical and microbial species-level features to the prediction of mortality. Each point represents one participant and indicates the magnitude and direction of each feature's impact on model output.

b, Qualitative assessment of the relative importance of features in predicting mortality, as estimated by an XGBoost model.

These analyses were based on 2,081 participants. † Species that remained statistically significant after adjustment for gene richness. PPI, proton pump inhibitor; BMI, Body Mass Index; SHAP, Shapley Additive Explanation Values. Source data are provided as a Source Data file.
