# Supplementary material for: Gut microbiota associates with frailty in older women
Source: Nat Commun. 2026 Jul 8;17:5925. doi: 10.1038/s41467-026-75176-5 (PMC13346421; doi:10.1038/s41467-026-75176-5)
Supplement: Supplementary file 2 — Description of Additional Supplementary Files [file 41467_2026_75176_MOESM2_ESM.pdf]

Description of Additional Supplementary Files

Supplementary Data 1: Microbial species significantly associated with the Frailty Mortality Index after correction for multiple comparisons (Bonferroni-adjusted), before and after adjustment for Gene Richness.
Associations between microbial species and the Frailty Mortality Index were assessed using linear regression models. P-values were derived from two-sided tests and adjusted for multiple comparisons using Bonferroni correction based on the number of analyzed species (n = 2,057), corresponding to a significance threshold of $P < 2.43 \times 10^{-5}$. Green P-values indicate statistical significance after Bonferroni correction. Analyses were performed before and after adjustment for gene richness and were based on 2,081 participants. † Species that remained statistically significant after adjustment for gene richness; ‡ unique species identified after gene richness adjustment.

Supplementary Data 2: Comparison of microbial species significantly associated with the Frailty Mortality Index (FMI) and Charlson Comorbidity Index (CCI) after correction for multiple comparisons (Bonferroni-adjusted).
Associations between microbial species and the Frailty Mortality Index (FMI) or Charlson Comorbidity Index (CCI) were assessed using linear regression models. P-values were derived from two-sided tests and adjusted for multiple comparisons using Bonferroni correction based on the number of analyzed species (n = 2,057), corresponding to a significance threshold of $P < 2.43 \times 10^{-5}$. Green P-values indicate statistical significance after Bonferroni correction. Analyses were performed before and after adjustment for gene richness and were based on 2,081 participants. † Species that remained statistically significant after adjustment for gene richness.

Supplementary Data 3: Sensitivity analysis of microbial species significantly associated with the Frailty Mortality Index, adjusted for comorbidities.
Associations between microbial species and the Frailty Mortality Index (FMI) were assessed using linear regression models adjusted for comorbidities. P-values were derived from two-sided tests, and false discovery rate (FDR)-adjusted q-values are reported to account for multiple testing. Comorbidities included rheumatoid arthritis, dementia, ischemic heart disease, heart failure, cerebrovascular disease, chronic pulmonary disease, liver disease, diabetes mellitus, renal failure, hemiplegia or paraplegia, peptic ulcer disease, solid non-metastatic tumor, lymphoma or leukemia, hyperparathyroidism, hyperthyroidism, and malnutrition. Analyses were based on 2,081 participants. ★ Species that remained significantly associated with FMI after adjustment for comorbidities.

Supplementary Data 4: Stratified analyses of FMI–microbiome associations by PPI, statin, and metformin use (users vs. non-users) after Bonferroni correction in the main analysis.
Associations between microbial species and the Frailty Mortality Index (FMI) were assessed using linear regression models separately among users and non-users of proton pump inhibitors

(PPIs), statins, and metformin. P-values were derived from two-sided tests. Overall analyses were based on 2,081 participants. Sample sizes for each stratum were: metformin non-users (n = 1,980) and users (n = 101); PPI non-users (n = 1,796) and users (n = 285); and statin non-users (n = 1,562) and users (n = 519).

Supplementary Data 5: Associations between clinical outcomes and microbial species positively associated with the Frailty Mortality Index after Bonferroni correction in the main analysis.
Associations between clinical outcomes and microbial species positively associated with the Frailty Mortality Index (FMI) were assessed using linear regression models adjusted for age, BMI, education level, smoking, alcohol intake, statin use, proton pump inhibitor use, and metformin use. P-values were derived from two-sided tests, and false discovery rate (FDR)-adjusted q-values are reported to account for multiple testing. The microbial species included in this analysis were limited to those positively associated with FMI and statistically significant after Bonferroni correction for multiple comparisons in the main FMI analysis. Analyses were based on 2,081 participants. † Species that remained statistically significant after adjustment for gene richness.

Supplementary Data 6: Associations between incident hip fracture, falls, death and microbial species positively associated with the Frailty Mortality Index after Bonferroni correction in the main analysis.
Associations between microbial species positively associated with the Frailty Mortality Index (FMI) and the risk of incident death, hip fracture, other fracture outcomes, and fall injuries were assessed using Cox proportional hazards models. Hazard ratios (HRs) and 95% confidence intervals are reported. P-values were derived from two-sided tests, and false discovery rate (FDR)-adjusted q-values are reported to account for multiple testing in the survival analyses. Models were adjusted for age, BMI, education level, smoking, alcohol intake, statin use, proton pump inhibitor use, and metformin use. The microbial species included in this analysis were limited to those positively associated with FMI and statistically significant after Bonferroni correction for multiple comparisons in the main FMI analysis. Analyses were based on 2,081 participants. † Species that remained statistically significant after adjustment for gene richness.

Supplementary Data 7: Associations between clinical outcomes and microbial species negatively associated with the Frailty Mortality Index after Bonferroni correction in the main analysis.
Associations between clinical outcomes and microbial species negatively associated with the Frailty Mortality Index (FMI) were assessed using linear regression models adjusted for age, BMI, education level, smoking, alcohol intake, statin use, proton pump inhibitor use, and metformin use. P-values were derived from two-sided tests, and false discovery rate (FDR)-adjusted q-values are reported to account for multiple testing. The microbial species included in this analysis were limited to those positively associated with FMI and statistically significant

after Bonferroni correction for multiple comparisons in the main FMI analysis. Analyses were based on 2,081 participants. † Species that remained statistically significant after adjustment for gene richness.

Supplementary Data 8: Associations between hip fracture, falls, death and microbial species negatively associated with the Frailty Mortality Index after Bonferroni correction in the main analysis.
Associations between microbial species negatively associated with the Frailty Mortality Index (FMI) and the risk of incident death, hip fracture, other fracture outcomes, and fall injuries were assessed using Cox proportional hazards models. Hazard ratios (HRs) and 95% confidence intervals are reported. P-values were derived from two-sided tests, and false discovery rate (FDR)-adjusted q-values are reported to account for multiple testing in the survival analyses. Models were adjusted for age, BMI, education level, smoking, alcohol intake, statin use, proton pump inhibitor use, and metformin use. The microbial species included in this analysis were limited to those negatively associated with FMI and statistically significant after Bonferroni correction for multiple comparisons in the main FMI analysis. Analyses were based on 2,081 participants. † Species that remained statistically significant after adjustment for gene richness.

Supplementary Data 9: Associations of gut metabolic modules (GMMs) with the FMI and risk of adverse outcomes.
Differential abundance of gut metabolic modules (GMMs) between participants with no frailty and severe frailty was assessed and is presented as log (fold change). Associations between GMMs and the Frailty Mortality Index (FMI) were assessed using Spearman correlation analyses and are presented as rho coefficients. Associations between GMMs and the risk of incident mortality, fall-related injuries, and hip fractures were assessed using Cox proportional hazards models and are presented as hazard ratios (HRs) with 95% confidence intervals (CIs). P-values were derived from two-sided tests, and false discovery rate (FDR)-adjusted q-values are reported to account for multiple testing within each analysis. Analyses were based on 2,081 participants.

Supplementary Data 10: Association between butyrate production potential and bacterial species positively associated with the Frailty Mortality Index after Bonferroni correction in the main analysis.
Associations between butyrate production potential and bacterial species positively associated with the Frailty Mortality Index (FMI) were assessed using linear regression models. Beta coefficients and 95% confidence intervals are reported. P-values were derived from two-sided tests, and false discovery rate (FDR)-adjusted q-values are reported to account for multiple testing in the butyrate production potential analyses. Models were adjusted for age, BMI, education level, smoking, alcohol intake, statin use, proton pump inhibitor use, and metformin use. The bacterial species included in this analysis were limited to those positively associated with FMI and statistically significant after Bonferroni correction for multiple comparisons in the

main FMI analysis. Analyses were based on 2,081 participants. † Species that remained statistically significant after adjustment for gene richness.

Supplementary Data 11: Association between butyrate production potential and bacterial species negatively associated with the Frailty Mortality Index after Bonferroni correction in the main analysis.

Associations between butyrate production potential and bacterial species negatively associated with the Frailty Mortality Index (FMI) were assessed using linear regression models. Beta coefficients and 95% confidence intervals are reported. P-values were derived from two-sided tests, and false discovery rate (FDR)-adjusted q-values are reported to account for multiple testing in the butyrate production potential analyses. Models were adjusted for age, BMI, education level, smoking, alcohol intake, statin use, proton pump inhibitor use, and metformin use. The bacterial species included in this analysis were limited to those negatively associated with FMI and statistically significant after Bonferroni correction for multiple comparisons in the main FMI analysis. Analyses were based on 2,081 participants. † Species that remained statistically significant after adjustment for gene richness.

Supplementary Data 12: The association between butyrate production potential and the risk of incident fracture, falls, and death.

Associations between butyrate production potential and the risk of incident death, hip fracture, other fracture outcomes, and fall injuries were assessed using Cox proportional hazards models. Hazard ratios (HRs) and 95% confidence intervals are reported per standard deviation (SD) decrease in butyrate pathway abundance. P-values were derived from two-sided tests. Models were adjusted for age, BMI, education level, smoking, alcohol intake, statin use, proton pump inhibitor use, and metformin use. Analyses were based on 2,081 participants.

Supplementary Data 13: Associations between measures of physical function, death and bacterial species in SUPERB and the Chinese Elderly cohort.

Associations between bacterial species and measures of physical function were assessed using linear regression models separately in the SUPERB and Chinese Elderly cohorts. Beta coefficients and 95% confidence intervals are reported. Associations with death were assessed using Cox proportional hazards models and are presented as hazard ratios (HRs) with 95% confidence intervals (CIs). P-values were derived from two-sided tests. Models were adjusted for age, BMI, education level, smoking, alcohol intake, statin use, proton pump inhibitor use, and metformin use. Analyses were based on 2,081 participants in the SUPERB cohort and 1,448 participants in the Chinese Elderly cohort. † Species that remained statistically significant after adjustment for gene richness; #N/A: taxa not identified in the Chinese Elderly cohort.
