# Supplementary material for: Gut microbiota associates with frailty in older women
Source: Nat Commun. 2026 Jul 8;17:5925. doi: 10.1038/s41467-026-75176-5 (PMC13346421; doi:10.1038/s41467-026-75176-5)
Supplement: Supplementary file 4 — Reporting Summary [file 41467_2026_75176_MOESM4_ESM.pdf]

nature portfolio

Corresponding author(s): Mattias Lorentzon

Last updated by author(s): Jun 2, 2026

Reporting Summary

Nature Portfolio wishes to improve the reproducibility of the work that we publish. This form provides structure for consistency and transparency in reporting. For further information on Nature Portfolio policies, see our Editorial Policies and the Editorial Policy Checklist.

Statistics

For all statistical analyses, confirm that the following items are present in the figure legend, table legend, main text, or Methods section.

n/a	Confirmed	
☐	☒	The exact sample size (*n*) for each experimental group/condition, given as a discrete number and unit of measurement
☐	☒	A statement on whether measurements were taken from distinct samples or whether the same sample was measured repeatedly
☐	☒	The statistical test(s) used AND whether they are one- or two-sided<br>*Only common tests should be described solely by name; describe more complex techniques in the Methods section.*
☐	☒	A description of all covariates tested
☐	☒	A description of any assumptions or corrections, such as tests of normality and adjustment for multiple comparisons
☐	☒	A full description of the statistical parameters including central tendency (e.g. means) or other basic estimates (e.g. regression coefficient) AND variation (e.g. standard deviation) or associated estimates of uncertainty (e.g. confidence intervals)
☐	☒	For null hypothesis testing, the test statistic (e.g. *F*, *t*, *r*) with confidence intervals, effect sizes, degrees of freedom and *P* value noted<br>*Give P values as exact values whenever suitable.*
☒	☐	For Bayesian analysis, information on the choice of priors and Markov chain Monte Carlo settings
☒	☐	For hierarchical and complex designs, identification of the appropriate level for tests and full reporting of outcomes
☐	☒	Estimates of effect sizes (e.g. Cohen's *d*, Pearson's *r*), indicating how they were calculated

Our web collection on statistics for biologists contains articles on many of the points above.

Software and code

Policy information about availability of computer code

Data collection	No software was used in data collection
Data analysis	The Unified Human Gastrointestinal Genome (UHGG) version 2.0 catalog was used for taxonomic profiling. Sequencing reads were processed using fastq_quality_trimmer from the FASTX Toolkit, Bowtie2 v2.4.4, Kraken2 v2.1.2 and Bracken v2.6.2. Batch correction was performed using metadeconfoundR. Gene counts were estimated using MEDUSA. Differential abundance analyses were performed using DESeq2 and ANCOM-BC. Gut metabolic modules were summarized using Omixer-RPM. Statistical analyses were performed in R v4.1.0 using the vegan package v2.6-6.1 and survival package v3.5-5. Machine-learning analyses were performed using the XGBoost R package v1.6.0.1 for FMI analyses and v1.7.5 for mortality analyses. The analysis code is openly available at: DOI: 10.5281/zenodo.20489792

For manuscripts utilizing custom algorithms or software that are central to the research but not yet described in published literature, software must be made available to editors and reviewers. We strongly encourage code deposition in a community repository (e.g. GitHub). See the Nature Portfolio guidelines for submitting code & software for further information.

April 2023

Data

Policy information about availability of data

All manuscripts must include a data availability statement. This statement should provide the following information, where applicable:

- Accession codes, unique identifiers, or web links for publicly available datasets
- A description of any restrictions on data availability
- For clinical datasets or third party data, please ensure that the statement adheres to our policy

The whole-metagenome sequencing data generated in this study have been deposited in the European Nucleotide Archive (ENA) database under study accession code PRJEB110772 [https://www.ebi.ac.uk/ena/browser/view/PRJEB110772] with public access. De-identified individual participant data are deposited in Figshare50. Other clinical data collected from this study will be available under restricted access for sensitive personal data protection, ethical restrictions, the General Data Protection Regulation (GDPR), and the Swedish Public Access to Information and Secrecy Act (SFS 2009:400); access can be obtained by submitting a data access request to Jan Boren (jan.boren@wlab.gu.se), head of the Institute of Medicine, Sahlgrenska Academy, University of Gothenburg, Gothenburg, Sweden. The RLAS cohort sequence data are available in the National Center for National Omics Data Encyclopedia under accession number code OEP001391 [https://www.biosino.org/node/project/detail/OEP001391]. Source data are provided with this paper.

Research involving human participants, their data, or biological material

Policy information about studies with human participants or human data. See also policy information about sex, gender (identity/presentation), and sexual orientation and race, ethnicity and racism.

Reporting on sex and gender	The SUPERB study included only postmenopausal women aged 75–80 years, recruited from the Swedish national population registry. As such, the study findings apply exclusively to one sex (female). Gender identity was not explicitly collected; participants were assumed to identify as women based on registry data and inclusion criteria. Sex was determined based on registry records (biological sex assigned at birth).<br>Consent was obtained from all participants for collection, analysis, and sharing of clinical and biological data, including stool samples for microbiota analyses. Sex-specific analyses were inherent to the study design, as only women were included. No comparative analyses by sex were performed, as men were not enrolled.<br>Justification for lack of sex- and gender-based analyses: The cohort was intentionally restricted to postmenopausal women to focus on the high-risk population for osteoporosis and bone fractures. Analyses by sex or gender were therefore not applicable. The RLAS cohort used for replication included both males and females (DOI: 10.1038/s43587-024-00678-0).
Reporting on race, ethnicity, or other socially relevant groupings	The SUPERB study did not explicitly collect data on race or ethnicity. Eligibility criteria required participants to be from the Swedish population and to have sufficient understanding of Swedish. The cohort consisted of women living in the Gothenburg area, Sweden, recruited via the Swedish national population registry.<br>Data on highest achieved education were retrieved from the Education Registry, Statistics Sweden, and categorized into predefined levels (e.g., primary, secondary, tertiary education). These variables were not used as a substitute for race or ethnicity.<br>Potential confounding factors were controlled in statistical models by including age, education level, BMI, smoking, alcohol intake, use of statins, proton pump inhibitors, metformin, and gene richness as covariates.<br>No other socially relevant groupings were used in the analyses.<br>In the RLAS cohort used for replication, all participants were Chinese (DOI: 10.1038/s43587-024-00678-0).
Population characteristics	The study cohort consisted of women aged 75–80 years from the SUPERB study with complete clinical and metagenomic data (n = 2,081), who were followed for a median of 7.9 years (interquartile range, 7.1–8.6). The Frailty Mortality Index (FMI), a composite score designed to capture frailty-related mortality risk, was developed in a larger sample (n = 3,028). More details on the characteristics of the participants included in this manuscript are provided in Table 1 and Figure 1. Findings were validated in an independent elderly Chinese cohort of 1,448 men and women aged 62-96 years (DOI: 10.1038/s43587-024-00678-0).
Recruitment	SUPERB participants were randomly selected from the Swedish national population registry. Invitations to participate were sent by letter and followed up by telephone between March 2013 and May 2016. Potential sources of bias include the restriction of the cohort to women who were ambulatory and able to communicate in Swedish, which may limit the generalizability of the findings to women with more severe mobility limitations or language barriers. There was no potential for self-selection bias among the study participants.<br>The RLAS is a community-based prospective cohort of older adults residing in rural areas of Rugao city, Jiangsu, China. Participants were recruited between November and December 2014 and are re-contacted every 1.5 years. Before the first field survey, elderly individuals were randomly selected according to 5-year age and sex strata, and equally across the 31 villages of Jiang'an township, based on the detailed registry of the Public Health Bureau of Jiang'an township. There was no potential for self-selection bias among the study participants (DOI: 10.1038/s43587-024-00678-0).
Ethics oversight	The study protocol for the SUPERB cohort was reviewed and approved by the Regional Ethical Review Board in Gothenburg, Sweden. All participants provided written informed consent prior to participation.<br>The RLAS cohort study was approved by the Ethics Committee of the Fudan University School of life sciences (No. BE1815). All participants provided written informed consent.

Note that full information on the approval of the study protocol must also be provided in the manuscript.

Field-specific reporting

Please select the one below that is the best fit for your research. If you are not sure, read the appropriate sections before making your selection.

- [x] Life sciences
- [ ] Behavioural & social sciences
- [ ] Ecological, evolutionary & environmental sciences

For a reference copy of the document with all sections, see nature.com/documents/nr-reporting-summary-flat.pdf

Life sciences study design

All studies must disclose on these points even when the disclosure is negative.

Sample size	No sample size calculations were performed for the SUPERB cohort, as the primary objective was exploratory, aiming to uncover microbiome associations in Swedish older women. The sample size was determined by the availability of fecal samples.<br>Similarly, no formal sample size calculations were conducted for the RLAS cohort, given its exploratory objective of investigating microbiome associations. The sample size was determined by the availability of fecal samples.
Data exclusions	For the SUPERB cohort, data were excluded only for participants lacking parameters required to calculate the frailty-mortality index, those without stool samples, and those who had used antibiotics within three months prior to stool collection. Beyond these exclusions, no additional data were omitted from the analyses.<br>For the RLAS cohort, participants were excluded from the metagenomic analysis if they had a history of gastrointestinal surgery, had used antibiotics prior to sampling, did not provide a fecal sample, or had insufficient metagenomic sequencing depth.
Replication	We have added information describing the measures taken to assess reproducibility. Briefly, FMI-associated species identified in the SUPERB cohort were tested for association with physical function parameters and mortality in an independent Chinese elderly cohort. Among the top 5% species most strongly associated with FMI, 11 species (55%) showed significant associations in the same direction in the replication cohort. Overall, 52.3% of all FMI-associated species showed concordant associations with at least one physical function measure and/or mortality. Thus, not all associations were replicated. Direct replication of FMI associations was not possible because the Chinese cohort lacked the data required to calculate the FMI.
Randomization	Randomization was not applicable because this was an observational population-based cohort study, not an interventional or randomized experimental study. Participants were not randomly allocated to experimental groups. FMI groups were defined after data collection based on the calculated Frailty Mortality Index. Potential confounding was addressed by adjustment for prespecified covariates in the statistical models, including age, BMI, smoking, alcohol intake, education level, medication use and, where applicable, gene richness and comorbidities.
Blinding	Blinding was not applicable because this was an observational population-based cohort study without treatment allocation or experimental intervention. Data collection followed standardized cohort protocols, and microbiome sequencing and bioinformatic processing were performed using predefined computational pipelines. Statistical analyses were based on prespecified models using objectively measured outcomes and covariates; therefore, investigator blinding to group allocation was not relevant.

Reporting for specific materials, systems and methods

We require information from authors about some types of materials, experimental systems and methods used in many studies. Here, indicate whether each material, system or method listed is relevant to your study. If you are not sure if a list item applies to your research, read the appropriate section before selecting a response.

Materials & experimental systems

n/a	Involved in the study	
x		Antibodies
x		Eukaryotic cell lines
x		Palaeontology and archaeology
x		Animals and other organisms
x		Clinical data
x		Dual use research of concern
x		Plants

Methods

n/a	Involved in the study	
x		ChIP-seq
x		Flow cytometry
x		MRI-based neuroimaging

Plants

Seed stocks	This study did not involve plants.
Novel plant genotypes	This study did not involve plants.
Authentication	This study did not involve plants.
