# Supplementary material for: Gut microbiota associates with frailty in older women
Source: Nat Commun. 2026 Jul 8;17:5925. doi: 10.1038/s41467-026-75176-5 (PMC13346421; doi:10.1038/s41467-026-75176-5)
Supplement: Supplementary file 5 — Transparent Peer Review file [file 41467_2026_75176_MOESM5_ESM.pdf]

nature portfolio

Peer Review File

Gut microbiota associates with frailty in older women

Corresponding Author: Professor Mattias Lorentzon

This file contains all reviewer reports in order by version, followed by all author rebuttals in order by version.

Version 0:

Reviewer comments:

Reviewer #1

(Remarks to the Author)
I appreciate that the revisions done by the authors have helped to substantially improve the quality of the manuscript from its earlier version (where it seemed that the authors' knowledge on the field especially in terms of the gut microbiome alterations associated with frailty was clearly limited). While I appreciate the thorough refinement of the manuscript on these lines, some of my comments pertaining to the methodology and the accessibility of the data still remain. I have summarized these below and will appreciate if the authors address it in a revision.

1. To my earlier comment "Ideally the authors should adopt a 3-fold or 2-fold cross-validation strategy for the same. But this was not clear in the manuscript." The authors have commented: "The Cox proportional hazards model was used for the derivation of the Frailty Mortality Index (FMI), to identify and weight the individual factors most strongly associated with mortality risk within the SUPERB cohort, leading to the creation of a composite index that captures frailty-associated mortality burden. Because it integrates several comprehensive measures that are specific to the SUPERB cohort, it cannot be directly reconstructed in other cohorts, which often lack these parameters. We therefore tested the performance of the FMI score in comparison to the Charlson Comorbidity Index (CCI) which is a well validated and widespread index used to characterize frailty. FMI performed consistently better in predicting frailty associated outcomes such as mortality, injurious falls and hip fracture during follow-up." I fully agree, but the validation has to be done in a manner where the values being reported are those where a given microbiome was not used for training. There are many ways this can be done. May be the authors are already doing this, but that has to be clarified. The authors cannot report training accuracies here in. It has to be either leave-one-observation-cross-validation (LOOCV) or fold-based cross validation. It is not clear how the authors did so.

2. My previous comments:
- "Continuing from my previous comments, many of the associations of gut microbes that the authors have been associated with frailty and age-associated health decline across multiple previous studies (10.1038/s43587-022-00306-9; 10.1038/s41575-022-00605-x; 10.7554/eLife.50240; 10.1126/science.aac8469) in addition to the Chinese cohort of Pu et al that they have used (https://doi.org/10.1038/s43587-024-00678-0). I would suggest two additional information that would greatly improve putting the current findings in the context of the current knowledge on gut microbiome and frailty"
- "While a lot of previous studies have reported taxa associations, very few have investigated the functional aspect. I would have loved to see the authors expand the gene-families linked to FMI and incidences of other frailty-associated adverse events and investigated the metabolic associations of these associated taxa (Pu et al have metabolomic data as well; there are other studies with matched gut microbiome and metabolomic data in elderly)."

The authors have reported literature-based insights. I want an actual investigation of the taxa identified in their study in terms of their metabolomic associations in other cohorts where such profiling was already done like Pu et al.

3. These kind of studies are not unique. Many such studies have been reported. To actually enable researchers appreciate the FMI index and use it in their studies and research, the authors have to make their sequencing data and metadata especially FMI, age, gender, publicly available (in deanonymized format). I would suggest the manuscript should not be accepted until they do so.

Reviewer #2

(Remarks to the Author)
The authors have successfully addressed my concerns, and I have no additional questions.

Reviewer #3

(Remarks to the Author)
I appreciate the authors' efforts to improve this manuscript. However, I have some additional comments:

The authors' response acknowledges that the FMI was designed as a "composite research index" rather than a clinical assessment tool, yet the manuscript persistently position it as a meaningful frailty measure. The authors' claim that the FMI "performed consistently better" than the Charlson Comorbidity Index (CCI) is not a rigorous benchmark because the CCI was designed to quantify comorbidity burden, not to capture multi-domain frailty. A more appropriate comparison would have been against the Rockwood Frailty Index or Fried Phenotype directly in predicting clinical outcomes. The authors should either provide this comparison or acknowledge that the superiority of the FMI has not been demonstrated against a comparable measure.

The authors' response to the request for strain-level analysis is notably deflected, redirecting to citations in the literature rather than conducting any new analysis. They state that several key species "have been reported previously in association with frailty," but this is precisely the point that was raised. Without strain-level resolution, it is unclear whether the same functional lineages are being detected across cohorts. The response acknowledges this limitation only generically ("a general methodological limitation of the field") without adequately engaging with what this means for the mechanistic conclusions drawn in the paper. Given that the authors use the UHGG v2 catalog, which provides genome-resolved species, strain-level assessment may have been more feasible than acknowledged.

Only 74% of associations remain nominally significant among medication users, and the authors attribute this to "reduced statistical power due to smaller sample sizes" without providing the actual stratum sizes. This explanation is insufficiently supported. If the medication-using subgroups are small, the power to detect real effects is also reduced, meaning the 74% figure may itself be an overestimate of robustness in a more adequately powered analysis. The authors should report the sample sizes for each medication stratum explicitly so that readers can evaluate whether statistical power, rather than true independence from medication effects, is driving the observed consistency.

The authors accept that their metabolic claims are inferred rather than observed, and acknowledge this in the Discussion. However, the response is minimal. If the authors acknowledge that these are predicted rather than measured metabolic activities, the language in the abstract and conclusions should be moderated accordingly.

The authors' response to the information leakage concern is partially satisfactory but leaves important details unresolved. However, if the feature selection was indeed performed within the training fold at each cross-validation iteration, the methodology is more defensible; however, if the 60 features were selected globally before cross-validation was initiated (as the original wording suggested), the concern remains valid. The authors should provide the exact order of operations and cross-validation architecture in sufficient detail to resolve this ambiguity definitively.

The authors' assertion that "replication was achieved with consistent directionality, strength, and statistical significance across cohorts" following β-coefficient inversion warrants scrutiny. Inverting coefficients to achieve apparent directional consistency is a post-hoc analytical decision that, while mathematically justifiable in cases of scale reversal, risks obscuring genuine heterogeneity between cohorts. The authors should report the raw pre-inversion data in supplementary materials and transparently document which specific tests required inversion and why, allowing readers to independently assess whether the "replication" is substantive or artifact of harmonization choices. Furthermore, the original concern that "the same species can have different functional capacities and dominant strains across geographically distinct populations" is only acknowledged but effectively dismissed as beyond scope. Given that SUPERB is a Swedish elderly cohort and the replication cohort is Chinese, the populations differ substantially in diet, lifestyle, medication patterns, and gut microbiome composition. This is not a peripheral methodological concern but a fundamental challenge to the generalizability of the FMI-microbiome associations, and it deserves more substantive engagement.

Version 1:

Reviewer comments:

Reviewer #1

(Remarks to the Author)
I thank the authors for the revisions made. All my suggestions and concerns have been addressed.

Reviewer #3

(Remarks to the Author)
I thank the authors for their revisions and for providing the additional analyses. Several points have been clarified, but my major concerns from the previous round remain insufficiently addressed. In several places, the rebuttal mainly documents limitations rather than resolving them, and some of the new disclosures actually reinforce my original concerns.

1. The authors acknowledge that no comparison with the Fried Frailty Phenotype or Rockwood Frailty Index was possible

and have added this as a limitation. I appreciate this textual revision, but simply acknowledging the limitation does not substitute for the validation required by the manuscript's current framing. The FMI is presented throughout as a frailty index, and the central claim that gut microbiota signatures track frailty depends on whether the FMI is actually capturing frailty, rather than a related but distinct construct. As noted previously, the comparison with the CCI is not sufficient for this purpose. If no data are available to benchmark the FMI against a validated frailty instrument, then the framing of the index should be reconsidered. At minimum, the abstract and conclusions should explicitly state that the index has not been validated against established frailty measures. The current revision mentions this only briefly in the Discussion, which is not adequate given how prominently the frailty framing appears in the abstract and headline claims.

2. Regarding strain-level resolution, the authors state that MAG-based strain analyses across more than 2,000 samples would require substantial computational resources and are beyond the scope of the present study. I understand this resource argument. However, there are less computationally intensive approaches to strain-level analysis that do not necessarily require de novo assembly or a MAG-based workflow. More importantly, the cross-cohort generalizability claim—that findings from the Swedish SUPERB cohort replicate in a Chinese cohort—is exactly the type of claim for which species-level resolution can be misleading. The manuscript's mechanistic and translational language has not been sufficiently revised to reflect this limitation. If strain-level analysis is out of scope, then claims about specific functional pathways, such as butyrate production by named species, and about cross-population replication should be downplayed or removed from the abstract, results, and discussion.

3. Regarding medication stratification, the new summary table is a clear improvement,. The permutation analyses and reporting of stratum sizes substantially address my earlier concern, particularly the explicit acknowledgment that the metformin stratum is underpowered. However, two issues remain. First, the observation that medication users have systematically higher FMI than non-users is not a neutral finding. It indicates that medication use and the exposure of interest are confounded, and the stratified analyses are therefore comparing groups that differ substantially in underlying frailty status. The authors note this in passing but do not adjust the interpretive framework accordingly. Associations preserved within higher-FMI medication-user strata may still reflect frailty-driven microbial signals, rather than independence from medication effects. Second, the percentage of associations significant within the medication-user strata is reported alongside permutation-based enrichment, but the manuscript text continues to emphasize the higher "directionally consistent" figure. I would ask the authors to lead with the within-stratum significance proportions in the text, since "same direction" is a considerably weaker criterion than independent replication.

4. For the XGBoost analysis, the authors now explicitly confirm that "the top 60 taxa associated with FMI were identified in the full dataset … and selected as input features for the XGBoost model." This is the scenario I was concerned about. The cross-validated performance metrics are therefore optimistically biased, and the magnitude of this bias is not quantified. I appreciate that the XGBoost analysis is framed as supplementary and exploratory, but the resulting figures are still presented to readers as performance estimates. I would therefore request one of the following:

a) Re-run the XGBoost analysis with feature selection nested within each cross-validation fold. This is a standard nested cross-validation procedure and does not require new data.

b) If this is infeasible, remove the numeric performance estimates from the main text and figures, and retain only a qualitative statement about which features contribute most to the model.

5. In the cross-cohort replication, my second concern on the challenge of generalizing from a Swedish elderly cohort to a Chinese elderly cohort is again addressed by adding limitation text rather than by analysis. Given the centrality of the replication claim to the paper's contribution, I would expect, at minimum: a comparison of FMI distributions and microbiome composition between the two cohorts; an explicit list of the 14 replicated species with side-by-side effect sizes, rather than directionality alone; and an acknowledgment in the abstract that replication is at the species level only and may not extend to strain-level functional capacity.At minimum, the authors should provide a comparison of FMI distributions and microbiome composition between the two cohorts, present the 14 replicated species side-by-side with effect sizes, confidence intervals, and P-values rather than directionality alone, and acknowledge in the abstract that replication is at the species level only. Most importantly, language framing this as a successful biological or mechanistic replication should be removed throughout: what has been shown is directional concordance of species-level associations across two demographically and ecologically distinct populations, not evidence that the same strains, functions, or host-microbe interactions are operating in both.

Open Access This Peer Review File is licensed under a Creative Commons Attribution 4.0 International License, which permits use, sharing, adaptation, distribution and reproduction in any medium or format, as long as you give appropriate credit to the original author(s) and the source, provide a link to the Creative Commons license, and indicate if changes were made.
In cases where reviewers are anonymous, credit should be given to 'Anonymous Referee' and the source.
The images or other third party material in this Peer Review File are included in the article’s Creative Commons license, unless indicated otherwise in a credit line to the material. If material is not included in the article’s Creative Commons license and your intended use is not permitted by statutory regulation or exceeds the permitted use, you will need to obtain permission directly from the copyright holder.
To view a copy of this license, visit https://creativecommons.org/licenses/by/4.0/

Gothenburg 2026-04-22

Editor
Dr Javier Martinez-Vesga
Nature Communications

Our ref: NCOMMS-26-003560A-Z

Reviewers' comments:

Reviewer #1 (Remarks to the Author):

I appreciate that the revisions done by the authors have helped to substantially improve the quality of the manuscript from its earlier version (where it seemed that the authors' knowledge on the field especially in terms of the gut microbiome alterations associated with frailty was clearly limited). While I appreciate the thorough refinement of the manuscript on these lines, some of my comments pertaining to the methodology and the accessibility of the data still remain. I have summarized these below and will appreciate if the authors address it in a revision.

1. To my earlier comment "Ideally the authors should adopt a 3-fold or 2-fold cross-validation strategy for the same. But this was not clear in the manuscript." The authors have commented: "The Cox proportional hazards model was used for the derivation of the Frailty Mortality Index (FMI), to identify and weight the individual factors most strongly associated with mortality risk within the SUPERB cohort, leading to the creation of a composite index that captures frailty-associated mortality burden. Because it integrates several comprehensive measures that are specific to the SUPERB cohort, it cannot be directly reconstructed in other cohorts, which often lack these parameters. We therefore tested the performance of the FMI score in comparison to the Charlson Comorbidity Index (CCI) which is a well validated and widespread index used to characterize frailty. FMI performed consistently better in predicting frailty associated outcomes such as mortality, injurious falls and hip fracture during follow-up." I fully agree, but the validation has to be done in a manner where the values being reported are those where a given microbiome was not used for training. There are many ways this can be done. May be the authors are already doing this, but that has to be clarified. The authors cannot report training accuracies here in. It has to be either leave-one-observation-cross-validation (LOOCV) or fold-based cross validation. It is not clear how the authors did so.

Author reply:

We thank the reviewer for this important comment and for the opportunity to clarify our analytical approach. The FMI was internally validated using leave-one-out cross-validation (LOOCV), whereby the Cox model was refitted after excluding each participant in turn, and the resulting coefficients were used to calculate an out-of-sample FMI value for the held-out individual. The FMI demonstrated similar discrimination for mortality before and after LOOCV (C-index: 0.688 and 0.683, respectively). We have added a sentence describing the LOOCV procedure in the Methods section **[line 657-660].**
We would also like to emphasize that the gut microbiome data were not used in the development of the FMI. The FMI composite score was derived independently using clinical and functional

variables (e.g., physical function, medical history) within the SUPERB cohort to capture frailty-associated mortality risk. The gut microbiome data were subsequently analyzed in relation to the FMI score.

2. My previous comments:
- "Continuing from my previous comments, many of the associations of gut microbes that the authors have been associated with frailty and age-associated health decline across multiple previous studies (10.1038/s43587-022-00306-9; 10.1038/s41575-022-00605-x; 10.7554/eLife.50240; 10.1126/science.aac8469) in addition to the Chinese cohort of Pu et al that they have used (https://doi.org/10.1038/s43587-024-00678-0). I would suggest two additional information that would greatly improve putting the current findings in the context of the current knowledge on gut microbiome and frailty"
- "While a lot of previous studies have reported taxa associations, very few have investigated the functional aspect. I would have loved to see the authors expand the gene-families linked to FMI and incidences of other frailty-associated adverse events and investigated the metabolic associations of these associated taxa (Pu et al have metabolomic data as well; there are other studies with matched gut microbiome and metabolomic data in elderly)."
The authors have reported literature-based insights. I want an actual investigation of the taxa identified in their study in terms of their metabolomic associations in other cohorts where such profiling was already done like Pu et al.

Author reply:

We thank the reviewer for this important comment. We agree that investigating the functional relevance of taxa, including their associated gene families and metabolic profiles, is an important direction to better understand the biological mechanisms linking the gut microbiome to frailty and related adverse outcomes.
To address this point, we assessed the associations between gut metabolic modules (GMMs) and the FMI using Spearman correlation analysis, identifying 66 GMMs significantly associated with the FMI (q-value < 0.05). All GMMs showing significant differential abundance between participants with no frailty and those with severe FMI (Figure 4a) were also significantly associated with the FMI using Spearman correlation. Furthermore, 34% of these GMMs were associated with mortality and 11% with injurious falls. GMMs associated with FMI, mortality and injurious falls are highlighted in Fig. 4a (Red ✦, grey and yellow labels, respectively). Spearman correlation coefficients and Cox proportional hazards estimates for mortality and injurious falls are provided in Extended Data Table 9.

The SUPERB cohort does not include metabolomic data, which limits our ability to directly assess metabolite associations within our study population. While studies such as Pu et al. include matched gut microbiome and metabolomic data, they do not have the same frailty-associated outcomes examined in our study (e.g., injurious falls, and hip fractures). A direct cross-cohort integration of taxa–metabolite associations would be challenging and may be confounded by differences in study design, population characteristics, and analytical methodologies. In addition, we do not have access to metabolite analyses in the external Chinese elderly cohort, which are beyond the scope of the present study. Future studies integrating multi-omics data will be valuable to further elucidate these mechanisms.

3. These kind of studies are not unique. Many such studies have been reported. To actually enable researchers appreciate the FMI index and use it in their studies and research, the authors have to make their sequencing data and metadata especially FMI, age, gender, publicly available (in deanonymized format). I would suggest the manuscript should not be accepted until they do so.

Author reply:

We thank the reviewer for this important comment. We agree that data availability is essential to facilitate transparency, and reproducibility. We have made the metagenomic sequencing data available and deposited in European Nucleotide Archive under accession number PRJEB110772. The accompanying metadata (FMI, age, gender, as recommended) has been included in the Extended Table 14 in the revised manuscript.
The dataset from the RLAS cohort can be accessed at the National Center for National Omics Data Encyclopedia, accessible via the following accession number: sequence data: OEP001391.

Reviewer #2 (Remarks to the Author):

The authors have successfully addressed my concerns, and I have no additional questions.

Author reply:

We thank the reviewer for the positive evaluation of our study and are pleased that our revisions have satisfactorily addressed all concerns.

Reviewer #3 (Remarks to the Authors):

I appreciate the authors' efforts to improve this manuscript. However, I have some additional comments:

- The authors' response acknowledges that the FMI was designed as a "composite research index" rather than a clinical assessment tool, yet the manuscript persistently position it as a meaningful frailty measure. The authors' claim that the FMI "performed consistently better" than the Charlson Comorbidity Index (CCI) is not a rigorous benchmark because the CCI was designed to quantify comorbidity burden, not to capture multi-domain frailty. A more appropriate comparison would have been against the Rockwood Frailty Index or Fried Phenotype directly in predicting clinical outcomes. The authors should either provide this comparison or acknowledge that the superiority of the FMI has not been demonstrated against a comparable measure.

Author reply:

We agree and thank the reviewer for this constructive comment. Due to data limitations in the SUPERB-cohort, we were not able to compare the FMI to Rockwood Frailty Index or Fried Phenotype. As requested, we have now acknowledged this limitation in the discussion: “The

FMI applied in this study was developed to identify gut microbiota signatures associated with frailty-related traits and outcomes. However, owing to data limitations in the SUPERB cohort, we were unable to compare the FMI with established multidomain frailty measures, such as the Fried Frailty Phenotype[6] or the Rockwood Frailty Index[7].” **[lines 520-524].** We have also changed wording throughout the manuscript **[lines 60-61; line 96; line 152; lines 384-385; 686-390; legend to Figure 1]**.

- The authors' response to the request for strain-level analysis is notably deflected, redirecting to citations in the literature rather than conducting any new analysis. They state that several key species "have been reported previously in association with frailty," but this is precisely the point that was raised. Without strain-level resolution, it is unclear whether the same functional lineages are being detected across cohorts. The response acknowledges this limitation only generically ("a general methodological limitation of the field") without adequately engaging with what this means for the mechanistic conclusions drawn in the paper. Given that the authors use the UHGG v2 catalog, which provides genome-resolved species, strain-level assessment may have been more feasible than acknowledged.

Author reply:

We thank the reviewer for raising this important point and agree that strain-level heterogeneity is important, particularly across geographically distinct populations. We apologize if our previous response appeared to deflect from this issue.
Our study aimed to develop the FMI composite index and assess its association with microbiome features. Therefore, we used a consistent bioinformatic framework to determine species-level associations and verify them across cohorts, to ensure comparability.
We agree that strain-level analyses, including metagenome-assembled genome (MAG) reconstruction, could in principle be applied to our data. However, implementing such analyses across >2,000 samples would require substantial additional computational resources and reprocessing of all datasets, and would extend beyond the scope of the present study, which is focused on the development of the FMI and its association with microbiome features.
While our approach allowed the identification of robust population-level associations, we now acknowledge that it does not capture strain-level functional heterogeneity, which may limit mechanistic interpretation of the observed associations. Therefore, we have amended the discussion to explicitly reflect this limitation **[lines 529–545]**.

- Only 74% of associations remain nominally significant among medication users, and the authors attribute this to "reduced statistical power due to smaller sample sizes" without providing the actual stratum sizes. This explanation is insufficiently supported. If the medication-using subgroups are small, the power to detect real effects is also reduced, meaning the 74% figure may itself be an overestimate of robustness in a more adequately powered analysis. The authors should report the sample sizes for each medication stratum explicitly so that readers can evaluate whether statistical power, rather than true independence from medication effects, is driving the observed consistency.

Author reply:

We thank the reviewer for this comment. We have now clarified and highlighted the sample sizes in the Extended Table 4. The *N* for each stratum is indicated in the column headers of the corresponding stratified results, as well as in a summary table (right panel of Extended Table 4), to facilitate transparency and interpretation.
The number of significant species exceeded that expected by chance under permutation analyses (1,000 interactions) for PPI and statins (268 for PPI users and 280 for statin users; both empirical P = 0.001). Fewer associations were observed among metformin users (47 observed vs 21.9 expected; P = 0.074), which had the smallest sample size (n = 103), suggesting that statistical power may be limited in this subgroup. Despite this, a high proportion of associations remained directionally consistent across strata.
We have also included FMI values stratified by medication use. Across all medication classes, users exhibited higher FMI compared to non-users (Table 1 and Extended Data Table 4), indicating greater frailty in these groups (metformin: 0.48 vs. −0.05; PPI: 0.41 vs. −0.09; statins: 0.17 vs. −0.09).
These findings suggest that the lower proportion of nominally significant associations among medication users is likely influenced by smaller subgroup sizes, particularly for metformin users, alongside differences in underlying phenotype that may contribute to greater heterogeneity of effects within these strata.
We have revised the manuscript accordingly to better reflect these considerations **[lines 236-242].**

Summary. Stratified analyses and permutation (1,000 interactions)

Medication	Stratum	N	FMI	P-value FMI: users vs non-users	N species sig overall	N species same direction (%)	N species sig stratum (%)	Permutation mean (SD)	empirical P
**Metformin**	**Non-users**	1,972	-0.05 (0.98)	**<0.001**	419	419 (100%)	419 (100%)	20.5 (14.2)	0.001
**Metformin**	**Users**	103	0.48 (1.05)		419	335 (80%)	47 (11.2%)	21.9 (16.6)	0.074
**PPI**	**Non-users**	1,776	-0.09 (0.93)	**<0.001**	419	419 (100%)	419 (100%)	21.8 (14.9)	0.001
**PPI**	**Users**	299	0.41 (1.19)		419	418 (99.8%)	268 (63.9%)	20.8 (15.4)	0.001
**Statin**	**Non-users**	1,545	-0.09 (0.97)	**<0.001**	419	419 (100%)	419 (100%)	21.5 (16.8)	0.001
**Statin**	**Users**	530	0.17 (1.02)		419	419 (100%)	280 (66.8%)	21.1 (13.3)	0.001

- The authors accept that their metabolic claims are inferred rather than observed, and acknowledge this in the Discussion. However, the response is minimal. If the authors acknowledge that these are predicted rather than measured metabolic activities, the language in the abstract and conclusions should be moderated accordingly.

Author reply:

We agree that the metabolic data are inferred and not directly measured. We have clarified this point in the limitation paragraph: “Metabolic inferences were derived from microbial gene module profiles rather than direct metabolomic measurements. As such, the predicted alterations in SCFA, amino-acid, and lipid metabolism reflect potential functional capacity rather than measured metabolite concentrations.” **[lines 526-529].** We have revised the manuscript to moderate the language and clarify that these findings reflect predicted metabolic capacity based on genomic annotation. Terms implying direct function have been replaced with more appropriate phrasing, including “predicted to mediate SCFA production” **[lines 457–458]**, “predicted butyrate production” **[lines 461, 441, 454]**, and “GMMs depleted with higher FMI captured functions predicted to be involved in gut and metabolic health” **[lines 451–452]**.
We have also revised the language in the Abstract to ensure that metabolic findings are consistently described as predicted rather than directly measured.

- The authors' response to the information leakage concern is partially satisfactory but leaves important details unresolved. However, if the feature selection was indeed performed within the training fold at each cross-validation iteration, the methodology is more defensible; however, if the 60 features were selected globally before cross-validation was initiated (as the original wording suggested), the concern remains valid. The authors should provide the exact order of operations and cross-validation architecture in sufficient detail to resolve this ambiguity definitively.

Author reply:

We thank the reviewer for this important comment and agree that clarification of the feature selection and cross-validation procedure is essential. We apologize for any lack of clarity in our previous response.
Our main results are presented in Extended Data Table 1 and are based on Bonferroni-corrected associations between microbial species and FMI derived from adjusted linear regression models. The XGBoost analysis was included as supplementary (Extended Data) to explore the predictive contribution and relative importance of FMI-associated taxa, rather than to develop a new predictive model.
Given the high dimensionality of the UHGG catalog (2,057 taxa) relative to our sample size (N = 2,075), including all taxa in the model would substantially increase the risk of overfitting and the inclusion of weak or non-informative predictors. We therefore restricted the feature space by selecting the top 60 taxa associated with FMI.

The top 60 taxa associated with FMI were identified in the full dataset (Extended Data Table 1) and selected as input features for the XGBoost model. We acknowledge that performing feature selection prior to cross-validation may introduce a degree of information leakage and could lead to optimistic estimates of model performance. To address this, we have clarified the order of operations in the Methods and added this as a limitation of the modeling **[779-782; 793-796; 822-825].** We have also modified the wording in Results **[lines 316-366].**

- The authors' assertion that "replication was achieved with consistent directionality, strength, and statistical significance across cohorts" following β-coefficient inversion warrants scrutiny. Inverting coefficients to achieve apparent directional consistency is a post-hoc analytical decision that, while mathematically justifiable in cases of scale reversal, risks obscuring genuine

heterogeneity between cohorts. The authors should report the raw pre-inversion data in supplementary materials and transparently document which specific tests required inversion and why, allowing readers to independently assess whether the "replication" is substantive or artifact of harmonization choices. Furthermore, the original concern that "the same species can have different functional capacities and dominant strains across geographically distinct populations" is only acknowledged but effectively dismissed as beyond scope. Given that SUPERB is a Swedish elderly cohort and the replication cohort is Chinese, the populations differ substantially in diet, lifestyle, medication patterns, and gut microbiome composition. This is not a peripheral methodological concern but a fundamental challenge to the generalizability of the FMI-microbiome associations, and it deserves more substantive engagement.

Author reply:

We would like to clarify that the direction of the β coefficients for the corresponding measures in the Chinese cohort was inverted solely to harmonize measurement units and, in Figure 5, for visualization purposes, thereby ensuring consistent interpretation across panels despite differences in how physical function measures were recorded. The original β coefficients are reported in full in Extended Data Table 13, allowing independent assessment of the results.
We have explicitly clarified the rationale for this transformation, specifying which measures required inversion and why, and have added a direct reference to Extended Data Table 13 in the Figure 5 legend.
“Associations between bacterial species and clinical phenotypes, including walking speed, chair stand, timed up-and-go, and mortality, were evaluated in the SUPERB cohort (n = 2,075) and examined for replication in an independent Chinese elderly cohort (n = 1,448). Of the top 5% FMI-associated species from SUPERB, 14 were replicated in the Chinese cohort, showing consistent associations with physical function and/or mortality. In the SUPERB cohort, walking speed was measured in meters per second and chair stand performance as the number of repetitions completed in 30 seconds, where higher values reflect better physical function. In contrast, in the Chinese cohort, both walking speed and chair stand were measured in seconds, where higher values indicate poorer performance. To ensure consistency and facilitate visual interpretation, the direction of the β coefficients for these two measures in the Chinese cohort was inverted (i.e., positive values were converted to negative, and vice versa). This transformation was applied for visualization purposes only. The timed up-and-go test was measured in seconds in both cohorts; no transformation was applied, as higher values consistently indicate poorer performance. The original β coefficients are reported in full in Extended Data Table 13.” **[**Figure 5 legend; **lines 1218–1242].**
We agree that differences between the SUPERB (Swedish) and Chinese cohorts, including diet, lifestyle, medication use, and gut microbiome composition, may influence the generalizability of the observed associations. While the consistency in species-level directionality supports the robustness of these findings at a population level, we acknowledge that underlying strain composition and functional capacities may differ across populations. We have expanded the Discussion to explicitly include this limitation and its implications for interpretation **[lines 529–545].**

Response to the Reviewers:

Reviewer #3 (Remarks to the Author):

I thank the authors for their revisions and for providing the additional analyses. Several points have been clarified, but my major concerns from the previous round remain insufficiently addressed. In several places, the rebuttal mainly documents limitations rather than resolving them, and some of the new disclosures actually reinforce my original concerns.

1. The authors acknowledge that no comparison with the Fried Frailty Phenotype or Rockwood Frailty Index was possible and have added this as a limitation. I appreciate this textual revision, but simply acknowledging the limitation does not substitute for the validation required by the manuscript's current framing. The FMI is presented throughout as a frailty index, and the central claim that gut microbiota signatures track frailty depends on whether the FMI is actually capturing frailty, rather than a related but distinct construct. As noted previously, the comparison with the CCI is not sufficient for this purpose. If no data are available to benchmark the FMI against a validated frailty instrument, then the framing of the index should be reconsidered. At minimum, the abstract and conclusions should explicitly state that the index has not been validated against established frailty measures. The current revision mentions this only briefly in the Discussion, which is not adequate given how prominently the frailty framing appears in the abstract and headline claims.

Authors response:

As requested in the previous revision round ("The authors should either provide this comparison or acknowledge that the superiority of the FMI has not been demonstrated against a comparable measure."), we acknowledged in the revised manuscript that such comparison was not possible in the SUPERB cohort due to absence of data on the frailty instruments Fried Frailty Phenotype or the Rockwood Frailty Index.

In addition, we would like to emphasize again that our aim was not to develop and validate a superior clinical frailty instrument, *but to identify gut microbiota species associated with relevant frailty related clinical outcomes.* If FMI would have been the primary objective of the study, we

fully agree that direct comparison with established frailty measures, such as the Rockwood Frailty Index or Fried Phenotype, would have been required.

The Frailty Mortality Index was derived within the SUPERB cohort using 16 clinical and functional factors associated with frailty and mortality, including measures related to physical function and medical history. These variables were evaluated in relation to death using Cox proportional hazards models over a median follow-up of 7.9 years. FMI was internally validated (LOOCV) and demonstrated consistent performance to captures a clinically meaningful phenotype associated with mortality and adverse outcomes. The FMI therefore shares several conceptual and clinical components with established frailty frameworks, but it differs in that it incorporates prospective mortality risk as the outcome used for index derivation.

We firmly disagree that the absence of a direct comparison with Fried or Rockwood invalidates the use of the FMI in the present study.

2. Regarding strain-level resolution, the authors state that MAG-based strain analyses across more than 2,000 samples would require substantial computational resources and are beyond the scope of the present study. I understand this resource argument. However, there are less computationally intensive approaches to strain-level analysis that do not necessarily require de novo assembly or a MAG-based workflow. More importantly, the cross-cohort generalizability claim—that findings from the Swedish SUPERB cohort replicate in a Chinese cohort—is exactly the type of claim for which species-level resolution can be misleading. The manuscript's mechanistic and translational language has not been sufficiently revised to reflect this limitation. If strain-level analysis is out of scope, then claims about specific functional pathways, such as butyrate production by named species, and about cross-population replication should be downplayed or removed from the abstract, results, and discussion.

Authors response:

We agree that several strain-level approaches beyond MAG reconstruction could in principle be applied to metagenomic datasets. However, we believe it is important to distinguish between different levels of biological resolution provided by these methods. Currently available strain-level approaches remain largely reference-dependent and may capture only subsets of strain variation, whereas genome-resolved analyses based on MAG reconstruction are required to determine the actual genomic and functional content present within metagenomic datasets across populations

(Cell. 2024 Nov 14;187(23):6550-6565.e11. doi: 10.1016/j.cell.2024.09.019, Nat Microbiol (2026). https://doi.org/10.1038/s41564-026-02334-2, *Nat Biotechnol* **43**, 1348–1359 (2025). https://doi.org/10.1038/s41587-024-02412-y).

In the present study focusing on species-level associations complemented by analyses of microbiome functional potential, including gut metabolic modules (GMMs) and butyrate-related pathways, to identify biological features associated with the FMI frailty related outcomes. The study was therefore not designed as a mechanistic strain-level investigation, but as a large-scale epidemiological and functional analysis of microbiome features associated with a multidimensional frailty-related mortality phenotype and several frailty related outcomes. We have further clarified the aim of the study in the abstract and conclusions.

We have also revised the manuscript to limit mechanistic interpretation and to acknowledge the limitations regarding strain heterogeneity. We agree that strain-resolved analyses represent important future work for mechanistic understanding. However, we do not believe that such analyses are required for reporting species-level associations and functional potential linked to frailty related outcomes across cohorts.

3. Regarding medication stratification, the new summary table is a clear improvement. The permutation analyses and reporting of stratum sizes substantially address my earlier concern, particularly the explicit acknowledgment that the metformin stratum is underpowered. However, two issues remain. First, the observation that medication users have systematically higher FMI than non-users is not a neutral finding. It indicates that medication use and the exposure of interest are confounded, and the stratified analyses are therefore comparing groups that differ substantially in underlying frailty status. The authors note this in passing but do not adjust the interpretive framework accordingly. Associations preserved within higher-FMI medication-user strata may still reflect frailty-driven microbial signals, rather than independence from medication effects. Second, the percentage of associations significant within the medication-user strata is reported alongside permutation-based enrichment, but the manuscript text continues to emphasize the higher “directionally consistent” figure. I would ask the authors to lead with the within-stratum significance proportions in the text, since “same direction” is a considerably weaker criterion than independent replication.

Authors response:

We revised the text as requested to include within-stratum significant proportions: “The FMI–microbiome associations showed near-complete directional consistency (>99%) across medication strata, with 67%, 64% and 11% of associations remaining significant in statins, PPI, and metformin users respectively (Extended Data Table 4).” Many confounding factors may affect the associations between frailty related outcomes and gut microbiota features, since frail individuals in general have more comorbidity, more medications, and are different in many other aspects, which was the reason for including a large number of potential confounders in all associations performed, in order to statistically adjust for these differences. This approach is generally recommended in epidemiology studies. We have also provided stratified analysis by medication group as requested.

4. For the XGBoost analysis, the authors now explicitly confirm that “the top 60 taxa associated with FMI were identified in the full dataset … and selected as input features for the XGBoost model.” This is the scenario I was concerned about. The cross-validated performance metrics are therefore optimistically biased, and the magnitude of this bias is not quantified. I appreciate that the XGBoost analysis is framed as supplementary and exploratory, but the resulting figures are still presented to readers as performance estimates. I would therefore request one of the following:
a) Re-run the XGBoost analysis with feature selection nested within each cross-validation fold. This is a standard nested cross-validation procedure and does not require new data.
b) If this is infeasible, remove the numeric performance estimates from the main text and figures, and retain only a qualitative statement about which features contribute most to the model.

Authors response:
The concern raised and proposed XGBoost analysis (Option A) is infeasible given the high dimensionality of the UHGG catalog (2,057 taxa) relative to our sample size (N = 2,075), including all taxa in the model would substantially increase the risk of overfitting and the inclusion of weak or non-informative predictors. As requested, we have removed the numeric performance measures from this analysis in the figure and manuscript text. This has been replaced by a qualitative statement, as requested.

5. In the cross-cohort replication, my second concern on the challenge of generalizing from a

Swedish elderly cohort to a Chinese elderly cohort is again addressed by adding limitation text rather than by analysis. Given the centrality of the replication claim to the paper's contribution, I would expect, at minimum: a comparison of FMI distributions and microbiome composition between the two cohorts; an explicit list of the 14 replicated species with side-by-side effect sizes, rather than directionality alone; and an acknowledgment in the abstract that replication is at the species level only and may not extend to strain-level functional capacity. At minimum, the authors should provide a comparison of FMI distributions and microbiome composition between the two cohorts, present the 14 replicated species side-by-side with effect sizes, confidence intervals, and P-values rather than directionality alone, and acknowledge in the abstract that replication is at the species level only. Most importantly, language framing this as a successful biological or mechanistic replication should be removed throughout: what has been shown is directional concordance of species-level associations across two demographically and ecologically distinct populations, not evidence that the same strains, functions, or host-microbe interactions are operating in both.

Authors response:

We agree that comparisons between the Swedish SUPERB cohort and the Chinese elderly cohort require cautious interpretation. Therefore, we have revised the manuscript further to clarify that the replication analyses are limited to species-level concordance and cannot be interpreted as evidence of shared strains and functional lineages across populations.

Specifically, we revised the abstract, introduction, discussion, and conclusions to consistently state that replication was observed at only the species level, and we removed language implying mechanistic or biological replication across cohorts.

Regarding the presentation of consistent species-level taxa, the original β coefficients, confidence intervals, and P-values have already been reported in full in Extended Data Table 13 and visualized in Figure 5. We additionally revised the text and figure legends further to emphasize concordant associations.

The FMI was not reconstructed in the Chinese cohort, and therefore direct comparison of FMI distributions between cohorts and in relation to the overall gut microbiome composition is not feasible. The aim of the analyses in the Chinese cohort was to evaluate whether FMI-associated species showed concordant associations with physical function and mortality, i.e. known frailty

related phenotypes across cohorts analyzed using a harmonized bioinformatic framework. The revised text now reflects this aim.
